# QSAR Models for the Prediction of Dietary Biomagnification Factor in Fish

**DOI:** 10.3390/toxics11030209

**Published:** 2023-02-23

**Authors:** Linda Bertato, Nicola Chirico, Ester Papa

**Affiliations:** Department of Theoretical and Applied Sciences, University of Insubria, 21100 Varese, Italy

**Keywords:** QSAR, biomagnification, bioaccumulation, MLR, alternatives to animal testing, data quality

## Abstract

Xenobiotics released in the environment can be taken up by aquatic and terrestrial organisms and can accumulate at higher concentrations through the trophic chain. Bioaccumulation is therefore one of the PBT properties that authorities require to assess for the evaluation of the risks that chemicals may pose to humans and the environment. The use of an integrated testing strategy (ITS) and the use of multiple sources of information are strongly encouraged by authorities in order to maximize the information available and reduce testing costs. Moreover, considering the increasing demand for development and the application of new approaches and alternatives to animal testing, the development of in silico cost-effective tools such as QSAR models becomes increasingly important. In this study, a large and curated literature database of fish laboratory-based values of dietary biomagnification factor (BMF) was used to create externally validated QSARs. The quality categories (high, medium, low) available in the database were used to extract reliable data to train and validate the models, and to further address the uncertainty in low-quality data. This procedure was useful for highlighting problematic compounds for which additional experimental effort would be required, such as siloxanes, highly brominated and chlorinated compounds. Two models were suggested as final outputs in this study, one based on good-quality data and the other developed on a larger dataset of consistent Log BMF_L_ values, which included lower-quality data. The models had similar predictive ability; however, the second model had a larger applicability domain. These QSARs were based on simple MLR equations that could easily be applied for the predictions of dietary BMF_L_ in fish, and support bioaccumulation assessment procedures at the regulatory level. To ease the application and dissemination of these QSARs, they were included with technical documentation (as QMRF Reports) in the QSAR-ME Profiler software for QSAR predictions available online.

## 1. Introduction

Bioaccumulation is a fundamental property for understanding the fate of a substance in the environment and its behaviour in the presence of living organisms. In a regulatory context, high-quality information that describes properties, activities and the fate of chemicals are required to improve risk assessment procedures [1]. Among the metrics available to describe bioaccumulation, the biomagnification factor (BMF) is defined as an increase in the fat-adjusted concentration of a substance in organisms at succeeding trophic levels in the food chain [1] and can be quantified as:

$$BMF=\frac{C_{predator}}{C_{prey}}$$

where *C_predator_* and *C_prey_* are the concentration in the predator and the prey, respectively, in steady-state conditions.

According to the OECD guidelines [2], BMFs should be corrected on the basis of the lipid content both in the fish (which is strongly associated with the bioaccumulation of hydrophobic chemicals) and in the food (Annex 7 [2]). The *BMF_L_* is usually derived from the *BMF* by dividing the *BMF* by the lipid content of the fish (*L_Fish_*) and multiplying by the lipid content of the diet (*L_Diet_*) [3].


$$BMF_{L}=BMF*\frac{L_{Diet}}{L_{Fish}}$$

In the literature, it was demonstrated that BMFs increased with increasing lipid content in the diet unless the lipids in the organisms and in the diet assumed similar sorption capacities for neutral hydrophobic chemicals [3,4]. In order to remove the apparent variability, Gobas and collaborators [3] proposed an alternative normalization, which included the standardization of the BMF to the lipid content of fish by dividing the *BMF* in units of kilograms of diet per kilogram of fish by the lipid content of the fish *L_Fish_* (kilograms of lipid per kilogram of fish), and then multiplying by the lipid content of the fish of 0.05 kg lipid/kg fish.

The BMF_5%_ has the advantage of having the same units as the wet weight BMF of kilograms per diet per kilograms of fish (which are different from the BMF_L_, lipid-corrected units). Despite this advantage, Gobas and collaborators [3] concluded that normalizing the BMF to both the lipid content of the fish and the lipid content of the diet, as recommended in the OECD 305 guideline [2], provided a more realistic indicator of the potential for biomagnification in the environment. For this reason, the data used here to develop the QSAR models are based on the normalization of the lipid content in both the fish and the diet.

An alternative to the BMF measured in laboratory experiments is the BMF determined in field studies. The field-derived BMF is the ratio between the steady-state concentrations in the organism and in the diet of the organism determined from the field where the organism is exposed to chemicals in the air, water and diet [5]. The laboratory- and field-derived BMFs differ in the sense that the first involves the exposure of the predator to chemicals only from the diet, while the second considers the uptake from both the respiratory medium and the diet [6,7].

The criterion that is commonly used to indicate the capability of a chemical to biomagnify is a BMF greater than 1 [5,8,9,10,11]. The rate of biomagnification in different types of organisms (e.g., fish, mammals and birds) can differ due to the greater capability of higher trophic-level organisms to biotransform chemicals [5,12].

For decades, dietary bioaccumulation testing in fish was conducted for scientific and regulatory purposes. In 2012, the Organization of Economic Co-operation and Development (OECD) provided a revised guidance (Test Guideline 305 [2]) based on a systemic review of dietary bioaccumulation testing methods, literature and data.

Among all of the metrics used to assess bioaccumulation, the dietary BMF is considered one of the best to be used for this purpose [5,13]. According to REACH (Annex III), to assess bioaccumulation it is necessary to consider all the information derived from the uptake of a chemical from different routes and to focus the efforts on in silico and in vitro approaches. However, while several in silico QSAR models have been developed for the estimation of BCF [14,15,16,17], only few QSARs are available for the prediction of the biomagnification factor [9,18]. For instance, Fatemi and Baher [18] developed linear and non-linear QSAR models based on BMF data measured in the field for 42 PCBs.

Another work published by Grisoni and co-workers [9] reported linear and non-linear QSAR models developed on selected data for 214 compounds extracted from the Arnot and Quinn dietary BMF database [8]. In our study, the Arnot and Quinn database is used for the development of QSAR models based on multiple linear regression and validated according to the OECD guidance.

The first aim of this paper is to develop QSARs based on multiple modelling techniques, taking into account data quality scores as provided in the original literature [8]. This represents an interesting innovation compared to the literature models developed using the same dataset. Furthermore, we want to compare low-quality data endpoints with the predictions generated using good-quality data, taking into consideration the structural applicability domain of the model. This procedure allows for the identification of inconsistent low-quality data, which deviate from the expected trend based on the QSAR generated from good-quality data. Furthermore, it allows for the identification of groups of chemicals that fall outside the applicability domain of the models based on good-quality data.

The QSAR models presented in this paper represent valuable tools, compliant with regulatory requirements, that can be applied to support chemical risk assessment procedures and have been included in the freely available QSAR-ME Profiler software [19].

## 2. Materials and Methods

### 2.1. The Literature Dataset

The dataset of laboratory-based fish biomagnification data published by Arnot and Quinn [8] was used to perform this study. The literature dataset is composed of 846 BMF data records from several sources and covers a wide range of Log BMF values from −5.70 to 1.95.

The dataset includes heterogeneous organic chemicals such as PCBs, legacy organochlorine pollutants, PBDEs, pesticides and siloxanes, and covers a wide range of estimated Log K_ow_ between 0.80 and 22.71. Almost 60% of the compounds have an estimated Log K_ow_ greater than 6.

### 2.2. Regression Models Dataset

Only BMF values flagged in the original database [8] as lipid-normalized were included in the final dataset (BMF_L_). Moreover, categories based on data quality assigned in Arnot and Quinn [8] were used to group data used for the development of multiple linear regression (MLR) QSARs. These categories were assigned after an in-depth review of the dietary BMF_L_ data, based on methods described in OECD test guidelines 305 [2]. Three subsets were generated from these categories: Dataset 1 included only Log BMF_L_ data assigned to high- and medium-quality categories. Dataset 2 included only low-quality data (which was not used to develop QSARs). The final dataset, Dataset 3, was composed of Dataset 1 and only reliable values extracted from Dataset 2, based on the results of the QSAR modelling. Dataset 1 was initially composed of 154 substances, of which 92 and 74 chemicals were of high or medium quality, respectively. Multiple data of both high and medium quality (available for 12 compounds) were averaged to obtain individual values for a single molecular structure to coherently develop the QSARs. Dataset 2 (Table S2) included 121 molecules, for which only data classified as low quality were available. Dataset 3 included 152 substances from Dataset 1, in addition to 106 low-quality data belonging to Dataset 2. Datasets 1 and 3 are reported in Supplementary Materials, excluding the outliers found during the modelling procedures (Tables S1 and S3).

### 2.3. Data Curation and Calculation of the Molecular Descriptors

Molecules were represented as SMILES (Simplified Molecular Line Entry Systems), which are string notations commonly used to describe molecular structures, including information on connectivity among atoms. For the data curation step performed for structural information, the matching of a structure in terms of both the SMILES and the CAS number was checked using the CIR (Chemical Identifier Resolver) [20] node in KNIME software [21]. The SMILES were converted into canonical SMILES using OpenBabel software [22] prior to comparison. This procedure allowed for the detection of compounds with the same molecular structure but different SMILES strings. Non-matching structures (i.e., CAS corresponding to incorrect SMILES) were also identified. BMF data referred to uncertain molecular structures, stereoisomers or chemicals with no corresponding CAS, were all excluded from the originally dataset.

Chemical information encoded into the SMILES was extracted through the calculation of molecular descriptors, which are numerical variables quantifying different aspects of the structural information of a chemical. One-dimensional, two-dimensional and fingerprints descriptors were calculated using the PaDEL-Descriptors (version 2.21) software [23] using the SMILES strings as input.

The following settings were applied to run the calculation of the molecular descriptors: remove salts, detect aromaticity and standardize nitro groups. More than 2600 molecular descriptors and fingerprints were calculated in this study. However, for further analysis, constant or nearly invariant descriptors, and descriptors with a pairwise correlation above 98% were excluded in a pre-reduction step using QSARINS software [24]; thus, about 500 molecular descriptors were finally retained and entered the variable subset selection procedure performed using a genetic algorithm (GA).

Theoretically calculated logarithmic octanol–water partition coefficients generated using PaDEL-Descriptors (e.g., XlogP, MLogP and CrippenLogP) were manually excluded from the final descriptors matrix. In fact, generated by different algorithms, these values might be inconsistent, thus introducing further uncertainty in the QSAR models [25,26].

### 2.4. Multiple Linear Regression Models

Multiple linear regression (MLR) by means of ordinary least squares (OLS) was used to develop the QSARs presented in this study. Prior to developing the QSAR models, Log BMF datasets were split into training and prediction sets. The latter was used as an external validation set of the QSARs, which meant that chemicals were not included during the model development. Chemicals were sorted by increasing response, and one out of three was then included in the prediction set. Chemicals with the highest and lowest value of the response were always included in the training set only. After performing the splitting, molecular descriptors were further filtered by removing invariant or correlated descriptors as described in Section 2.3.

MLR-OLS QSARs were then developed using QSARINS software [24] by applying the all-subset procedure, followed by a genetic algorithm variable subset selection (GA-VSS) for variable selection [27].

### 2.5. Applicability Domain

The applicability domain of MLR models was studied by evaluating standardized residuals and the leverage calculated using the descriptors included in the model. Compounds with standardized residuals greater than 2.5 standard deviation units were flagged as response outliers while compounds with a leverage value (h*) larger than 3 p′/n (i.e., high-leverage chemicals), where p′ is the number of model variables incremented by 1 and n is the number of training objects, were flagged as out of the structural domain of the model [28]. The plot of hat values (h) versus standardized residuals was used for a graphical evaluation.

The datasets used to generate QSAR models in this study are reported as Supplementary Materials (Tables S1–S3).

## 3. Results and Discussion

The first dataset used to generate the models was Dataset 1, which included high- and medium-quality data for 154 structurally heterogeneous compounds, see Table S1. A QSAR model developed on such data should be highly reliable. This model was used to investigate the reliability of the low-quality data (Dataset 2), see Table S2, and to address the possible uncertainty associated with specific chemicals. A final model was developed including high-quality and consistent data (i.e., Dataset 1 pooled with reliable data from Dataset 2), see Table S3. The main aim of this study was to propose a new linear QSAR model for dietary BMF prediction, applicable to a wide range of organic compounds with different chemical structures.

### 3.1. Log BMF_L_ QSAR Based on Dataset 1

The first modelling attempt performed on Dataset 1 highlighted the presence of two recurrent outliers: 3,3’,4,5-Tetrachlorobiphenyl (CAS: 70362-49-1) with Log BMF_L_ −2.52 and Propiconazole (CAS: 60207-90-1) with Log BMF_L_ −2.22. These single experimental data were classified as medium quality. We wanted to highlight that the experimental data available for 3,3’,4,5-Tetrachlorobiphenyl was inconsistent if compared with the experimental values available for similar compounds in the dataset. For instance, the Log BMF_L_ reported for 3,3’,4,5-Tetrachlorobiphenyl was the lowest value in comparison to the experimental values available for other tetra-PCBs, whose values were mostly in the 0.22–0.80 range. Propiconazole had the lowest value in comparison to the other fungicides in the same category, whose Log BMF_L_ values were around −2.

These discrepancies might explain the limited accuracy of the QSAR predictions calculated for these two outliers, which were removed from Dataset 1. The outliers excluded from further modelling steps are listed in Table S4.

A training set of 115 compounds with a Log BMF_L_ range between −2.3 and 0.93 was then used to develop a new population of GA-selected MLR-QSARs based on Dataset 1. A five-fold cross-validation was used to check the internal predictivity of the models in the population, in addition to the quantification of the fitting (R^2^). The best model chosen from the GA population, externally validated on 37 chemicals, was based on 7 molecular descriptors:

(1) $${\mathrm{logBMF}}_{L}\left(\mathrm{dietary}\right)=-103.01\;\left(\pm 25.20\right)-\;0.08\;\left(\pm 0.02\right)AATS5i\;+\;9.74\;\left(\pm 2.03\right)BCUTw-1l\;-\;\;0.88\;\left(\pm 0.27\right)\;PubchemFP257\;-\;\;0.15\;\left(\pm 0.05\right)\;C3SP2\;+\;3.14\;\left(\pm 1.50\right)\;MATS1i\;-\;0.54\;\left(\pm 0.40\right)\;GATS5m\;+\;\;0.29\;\left(\pm 0.25\right)\;GGI5$$

n_training_ = 115; n_prediction_ = 37; R^2^ = 0.79; RMSE_tr_ = 0.41; Q^2^LOO = 0.76; Q^2^LMO = 0.75; RMSE cv = 0.44; MAE_tr_ = 0.32; RMSE_ext_ = 0.49; MAE_ext_ = 0.38; R^2^_ext_ = 0.68; Q^2^-F3 = 0.70; CCC_ext_ = 0.81.

Table 1 shows the averages of the RMSE_test_ and the MAE_test_ values of the models based on seven descriptors, developed for each fold of Dataset 1.

The average of the RMSE_test_ of the k-fold population was 0.80, which was higher but still comparable to the RMSE_ext_ (0.49) calculated for Equation (1). The difference between RMSE_ext_ and RMSE_tr_ might be due to the presence of outliers in the training set. In fact, the cross-validated and external MAE values, which were less sensitive to outliers, were more similar (MAE_test_ = 0.59 with respect to MAE_ext_ = 0.38).

**Figure 1:**
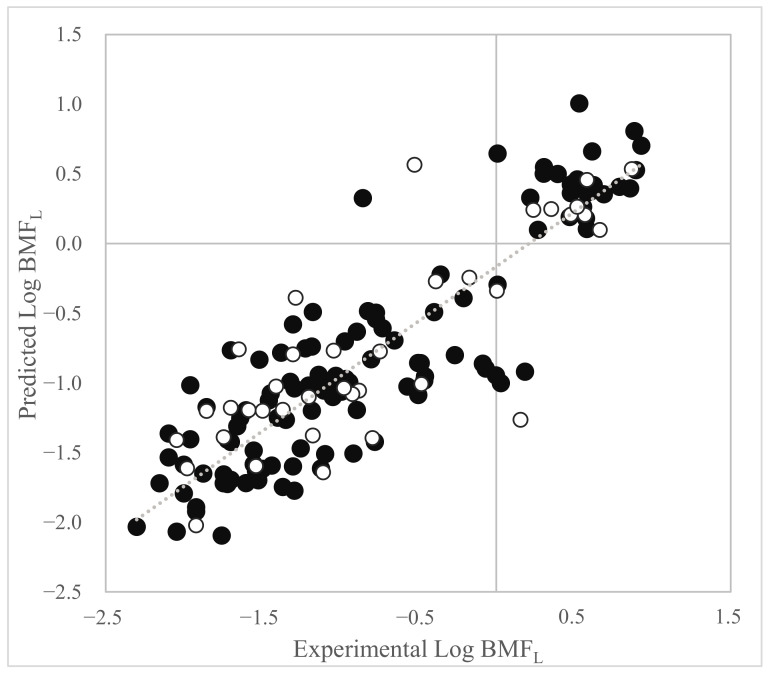
Plot of experimental vs. predicted Log BMF_L_ values (calculated by Equation (1)). Black dots = training set. White dots = prediction set.

The plot of the experimental versus predicted values for the model based on Dataset 1 is shown in Figure 1.

Furthermore, in the five-fold populations, GATS2i, SubFPC295, PubchemFP257, PubchemFP503 and PubchemFP738 were among the most frequently selected descriptors across the models of the seven variables. GATS2i was the Geary autocorrelation—lag 2/weighted by the first ionization potential, and the SubFPC295 was related to the presence of heteroatoms and counted how frequently the bonds between the C and O, N or S atoms were counted within the chemical structure. The binary fingerprint, PubchemFP257, was also present in Equation (1) and was related to the presence of two or more aromatic rings. PubchemFP503 and PubchemFP738 revealed the presence of different fragments, both containing the Cl atom.

The use of the k-fold supported the estimation of the predictivity of the model and confirmed which structural features were relevant to estimate the biomagnification potential.

Table 2 lists the molecular descriptors of Equation (1). In particular, the AATS5i descriptor was the most important variable selected in Equation (1) and was related to ionization potential. The presence of this feature in the molecular structure was inversely related to an increase in the values of dietary BMF_L_. The second most important molecular descriptor, BCUTw-1l, was encoding structural information related to molecular diversity based on the information extracted from the burden matrix [29,30]. In the literature, the same molecular descriptor was selected to develop classification models to predict biomagnification [31]. Furthermore, the presence of two or more aromatic rings (PubchemFP257) within the molecular structure led to an increase in the molecular weight and dimension, which seemed to decrease the bioaccumulative ability. In fact, this descriptor had a negative sign in the equation. A total of 84 chemicals in the dataset were characterized by the presence of 2 or more aromatic rings, while 68 chemicals did not have this fragment within their molecular structure. The descriptor, C3SP2, encoded for the presence of unsaturated branched aliphatic systems [32]. All the compounds with large C3SP2 values (greater than 5) were PAHs and had Log BMF values between −2.5 and −1. MATS1i and GATS5m were autocorrelation molecular descriptors and were related to ionization potential and molecular weight, respectively. Finally, GGI5 was a topological charge descriptor [30,33].

**Figure 2:**
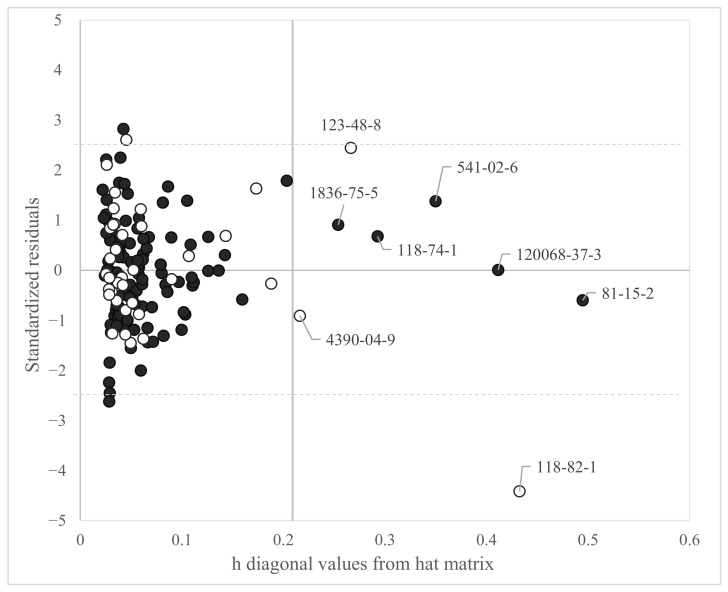
Applicability domain of the MLR-OLS model selected from the GA population (Equation (1)). The cut-off value on the abscissa for Equation (1) is h* = 0.209. Chemicals with h values larger than h* are outside the AD of the model. Black dots = training set. White dots = prediction set.

The applicability domain calculated for the model is reported in Figure 2.

Figure 2 shows that only a few chemicals lay far from the central space of the model (i.e., the space on the left of the horizontal cut-off value h* = 0.209). In particular, one chemical was highlighted as out of the structural AD and heavily out of the response AD of the model (CAS 118-82-1), i.e., it had standardized residuals larger than 2.5 standard deviation units. This molecule, called Binox M, was characterized by a large molecular structure. It was categorized as an antioxidant and used in fuel, polymers and lubricant blending industries, as well as an antioxidant additive in petroleum-based lubricants. The Log BMF for this chemical was predicted with a residual larger than 4 standard deviations. However, Binox M was in the prediction set and it did not influence the model development. Six other chemicals are highlighted in Figure 2, both for the training set and the prediction set, as out of the structural AD of the model (CAS: 81-15-2, 120068-37-3, 541-02-6, 1836-75-5 for the training set. CAS 118-74-1 and 4390-04-9 for the prediction set); however, were predicted by the model with standardized residuals within 3 standard deviations. The list and the molecular structure of the chemicals highlighted in Figure 2 are reported in Table S5.

The split model Equation (1) was recalibrated, pooling the training and the prediction set. The equation (Equation (2)) of the full model is reported below:

(2) $${\mathrm{logBMF}}_{L}\left(\mathrm{dietary}\right)=\;-103.72\;\left(\pm 22.93\right)-\;0.07\;\left(\pm 0.02\right)AATS5i\;+\;9.74\;\left(\pm 1.85\right)\;\;BCUTw-1l\;-\;\;0.83\;\left(\pm 0.25\right)\;PubchemFP257\;-\;0.15\;\left(\pm 0.05\right)\;C3SP2\;+\;3.73\;\left(\pm 1.36\right)\;MATS1i\;+\;0.42\;\left(\pm 0.21\right)\;GGI5\;-\;\;0.55\;\left(\pm 0.34\right)\;GATS5m$$

n_training_ = 152; R^2^ = 0.77; RMSE_tr_ = 0.43; Q^2^LOO = 0.74; Q^2^LMO= 0.73; RMSEcv = 0.46; MAE_tr_ = 0.33.

### 3.2. Application of the Model to Investigate Reliability of Data Identified as Low Quality (Dataset 2)

Possible inconsistencies between the experimental data categorized as low quality (Dataset 2) and the predictions based on Equation (2) were investigated as follows.

**Figure 3:**
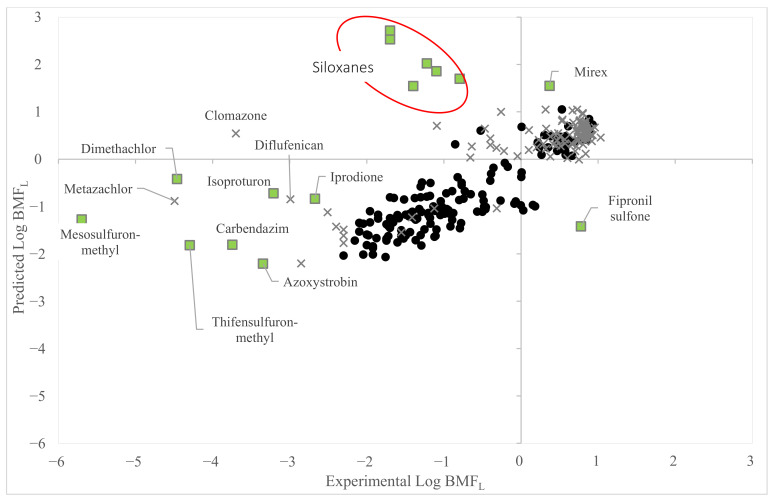
The figure shows the plot of the experimental (x-axis) vs. the predicted values (y-axis) of Equation (2), which are represented by black dots. Equation (2) is applied to predict remaining low-quality data. Data corresponding to low quality are here plotted with a grey “X” (data included in the AD) or squares (data excluded from the AD).

Figure 3 shows that most of the low-quality data fell in the AD of Equation (2) and had experimental values consistent with the predictions generated by this QSAR. Clomazone, Diflufenican and Metazachlor fell within the structural AD of the model, but they had large errors in prediction.

On the other hand, 15 chemicals fell outside the structural AD of the model (see the green squares in Figure 3). Among these, the experimental Log BMF_L_ values available for the pesticides were mostly consistent with the expected values predicted by Equation (2). However, the large deviations in the prediction from the experimental values were highlighted for siloxanes in the red ellipse on the top of Figure 3. Low-quality data falling outside the structural AD of Equation (2) were unreliable; therefore, they were not used to assess the consistency between the experimental Log BMF_L_ and the QSAR predictions. Furthermore, we wanted to point out that Equation (2) was not suitable to predict the Log BMF_L_ of siloxanes. This fact highlighted that additional experiments would be necessary to generate good-quality data for siloxanes. This would be useful to extend the AD of future models to these chemicals.

### 3.3. Log BMF_L_ QSAR Based on Dataset 3

A new model was developed using the final dataset, named Dataset 3, which combined Dataset 1 and only the reliable low-quality data (i.e., included in the AD of Equation (2)). The analysis of the best models in the population led to the identification of 15 chemicals repeatedly mispredicted or falling outside the structural AD of multiple models. These 15 outliers, in addition to 3,3’,4,5-Tetrachlorobiphenyl and Propiconazole (already excluded in Equation (1)), listed in Table S4, were not included in the next modelling steps.

The equation of the best model was based on seven molecular descriptors as follows:

LogBMF_L_ (dietary) = −0.90 (±0.19) + 1.41 (±0.19) *PubchemFP*503 − 0.40 (±0.07) *SubFPC*295 − 0.06 (±0.02)
*R_TpiPCTPC* + 0.56 (±0.23) *MLFER_S* − 1.39 (±0.60) *maxHother* + 0.65 (±0.26) *GGI*5 − 4.00 × 10^−3^ (±2.5 × 10^−3^) *VE*3_*Dt* (3)

n_training_ = 194; n_prediction_ = 64; R^2^ = 0.85; RMSE_tr_ = 0.43; Q^2^LOO = 0.83; Q^2^LMO = 0.82; RMSE cv = 0.45; MAE_tr_ = 0.33; RMSE_ext_ = 0.58; MAE_ext_ = 0.40; R^2^_ext_ = 0.73; Q^2^-F3 = 0.72; CCC_ext_ = 0.84.

Table 3 shows the results from the five-fold cross-validation performed on the training set and the averages of the RMSE_test_ and the MAE_test_ values of the models based on seven descriptors, developed for each fold:

The average RMSE_test_ of the five-fold cross-validation procedure listed in Table 3 was equal to 0.65, which was comparable to the RMSE_ext_ calculated for Equation (3) (0.58).

In this case, the analysis of the frequencies of the selected variables in the seven-size population of the five-fold cross-validation also showed that PubchemFP503 was selected in almost all the populations. Other frequent variables were SubFPC295 and nBondsS3, which represented the total number of single bonds (excluding the bonds to hydrogens and aromatic bonds), and the PubchemFP38, which was related to the presence of two or more chlorine atoms within the molecular structure.

**Figure 4:**
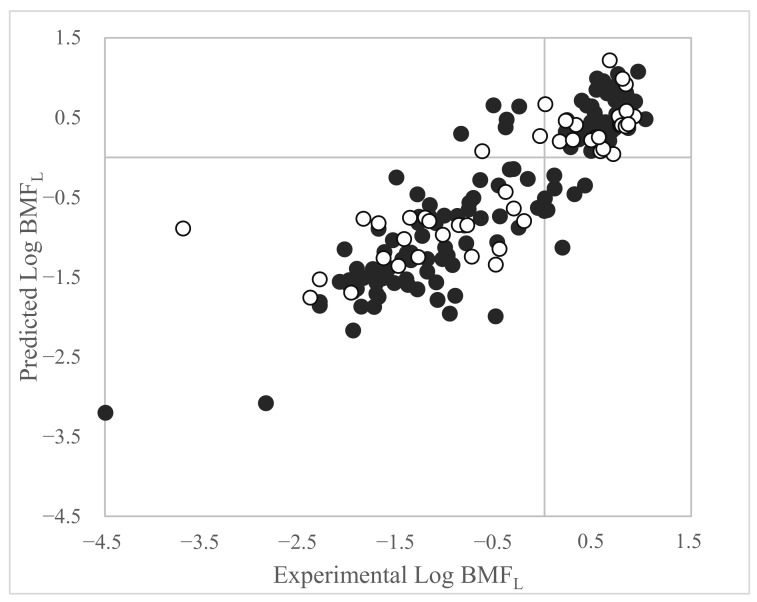
Plot of experimental vs. predicted Log BMF_L_ values of the proposed MLR-OLS model based on Dataset 3 (Equation (3)). Black dots = training set. White dots = prediction set.

The plot of the experimental versus predicted values for the model based on Dataset 3 is shown in Figure 4.

Table 4 includes a brief description of the meaning of the descriptors included in Equation (3), which are listed in decreasing order of importance.

In this model, the most important molecular descriptor was the binary fingerprint, PubchemFP503, followed by the substructure fingerprint count, SubFPC295. PubchemFP503 counted the presence of a simple SMARTS pattern, which considered the presence of a bond aromaticity, a chlorine atom and a triple bond. The SubFPC295 descriptor was related to the presence of heteroatoms and counted how frequently the bonds between the C and O, N or S atoms was counted within the chemical structure. The presence of these bonds within the molecular structures was inversely related to the values of the dietary BMF. These two fingerprints were often selected in the five-fold cross-validation population developed for Dataset 1. The other molecular descriptors were related to the topology and the size of the molecule, such as the R_TpiPCTPC and the GGI5 descriptors. The MLFER_S descriptor was related to polarizability. VE3_Dt was a 2D matrix-based descriptor obtained using the detour matrix [30]; the negative sign in the equation for VE3_Dt suggested a negative contribution to the activity.

**Figure 5:**
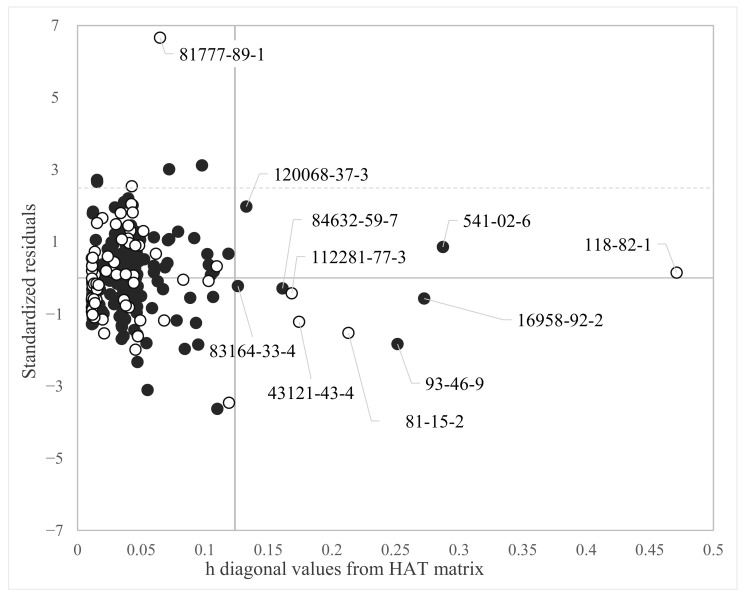
Applicability domain of Equation (3). The cut-off value on the abscissa for Equation (3) is h* = 0.1237. Values of h below h* are within the structural AD of the model. Black dots = training set. White dots = prediction set.

The applicability domain calculated for the model (Equation (3)) is reported in Figure 5.

Figure 5 highlights Binox M (CAS:118-82-1) as a high-leverage compound, which was already detected as both a structural and a response outlier in Equation (1). In this case, Binox M was instead correctly predicted by the model with a standardized residual close to zero.

Clomazone (CAS: 81777-89-1) was a strong outlier in the prediction set as in Equation (2).

The other chemicals highlighted in Figure 5 were structural high leverage but were correctly predicted by the model. The list and the molecular structure of the chemicals highlighted in Figure 5 are reported in Table S5.

Equation (3) was calibrated using the full dataset, and the corresponding best equation is:

(4) $${\mathrm{logBMF}}_{L}\left(\mathrm{dietary}\right)=\;-\;1.04\;\left(\pm 0.16\right)\;-\;0.42\;\left(\pm 0.06\right)\;SubFPC295\;+\;1.38\;\left(\pm 0.18\right)\;PubchemFP503\;-\;\;0.06\;\left(\pm 0.02\right)\;R\_TpiPCTPC\;+\;0.57\;\left(\pm 0.23\right)\;MLFER\_S\;+\;0.73\;\left(\pm 0.22\right)\;GGI5\;-\;1.05\;\left(\pm 0.55\right)\;maxHother\;–\;\;5.00\times 10^{-3}\;(\pm 2.4\times 10^{-3})\;VE3\_Dt$$

n_training_ = 258; R^2^ = 0.82; RMSE_tr_ = 0.47 Q^2^LOO = 0.80; Q^2^LMO = 0.80; RMSEcv = 0.49; MAE_tr_ = 0.35.

### 3.4. Comparison with Existing BMF_L_ QSAR Models

As was mentioned in the Introduction, few QSAR models were available for the prediction of the dietary BMF in fish [9,18]. Their performances were compared with our models developed in this study, as reported in Table 5.

The study published by Fatemi and Baher [18] proposed linear and non-linear QSAR models for the prediction of the Log BMF for a dataset including 30 polychlorinated biphenyl (PCB) congeners and 12 organochlorine pollutants. These models were hardly comparable with the QSARs developed here. In fact, they were trained on only 42 compounds, with rather homogeneous molecular structures, included up to 5 molecular descriptors, and covered an experimental range of Log BMFs smaller than the other models reported in Table 5. In general, the fitting performances were comparable across all the models reported in Table 5. However, not surprisingly, the literature model was more accurate (especially when ANN were used) to predict Log BMF for PCBs and similar compounds, whereas more general models, based on heterogeneous datasets, had larger RMSE_ext_.

A closer comparison could be conducted between the QSARs proposed here and the regression models published in Grisoni and colleagues [9], since data were taken from the same literature source [8]. However, we wanted to highlight that information related to lipid normalization of the BMF values was not considered by Grisoni et al. [9], and therefore the final values of Log BMF modelled in the two studies, as well as the structural and response domain of the models, were different.

In the work published by Grisoni et al. [9], a subset of 214 compounds was extracted from the Arnot and Quinn Log BMF database [8]. Two types of QSAR models were proposed with different levels of predictivity and interpretability. Model 1 (M1), developed using a weighted nearest-neighbour regression (wNNR), was based on four molecular descriptors calculated using DRAGON software. The molecular descriptors that were selected in wNNR were: the squared octanol–water partitioning coefficient (MlogP2), the total number of bonds (nBT), and two types of molecular substructures (B02[N-O] and F06[C-C]). The second model (M2) was an MLR model based on seven molecular descriptors: MlogP2, X0Av, X1Per, SaaaC, VE1_B(m), B02[N-O] and B03[N-Cl]. MlogP2 was the squared logarithm of the octanol–water partitioning coefficient (Log kow) and was selected in both the models. The other molecular descriptors were mainly related to the molecular size, the presence of heteroatoms (e.g., O, N, P), the presence of rings and the typology of the bonds. The predictions generated by M1 and M2 were then combined by consensus [9]. It was interesting to highlight that some of the structural features similar to those selected in models M1 and M2 were also selected in our QSARs. These included the general aspects related to molecular size and the presence of heteroatoms, as well as the fragments detecting the presence of chlorine atoms and aromatic rings, which were features related to molecular hydrophobicity. This supported the relevance of these descriptors for modelling dietary BMF. Moreover, the SubFPC295 descriptor was also identified in a former study as relevant for modelling Log BCF in fish [34].

Finally, the comparison of the external performances of the models of Grisoni and colleagues with those calculated here for Equations (1) and (3), showed that these models had similar predictivity when they were tested on chemicals not used to train the models.

These results also highlighted the importance of studies addressing the creation and curation of large databases [8,35,36,37], which are useful for the development of meaningful QSAR models.

## 4. Conclusions

Bioaccumulation is one of the PBT properties that authorities require to assess in the evaluation of the risks that chemicals may pose to humans and the environment.

In this work, a large curated database of fish laboratory-based BMF values available in the literature [8] was used to develop externally validated QSAR models following OECD guidance [38]. In contrast to previous studies, only data reported as lipid-normalized (Log BMF_L_) were used to create the models. Furthermore, data were combined according to data quality scores (high, medium and low) assigned in the original dataset on the basis of perceived data quality and consistency with the OECD guidelines [8].

Two regression models for the prediction of Log BMF_L_ were suggested as the final output in this study. One QSAR was based only on good-quality data (Dataset 1). This model was used to assess the consistency of low-quality data, and allowed for the identification of some problematic compounds, such as siloxanes and highly brominated and chlorinated compounds. Additional experimental effort is necessary to generate good-quality data for these chemicals. This will be useful in the future to train QSARs on better data and larger applicability domains, which will allow for the reliable prediction of similar compounds.

The other model, which was developed on a larger structural and response domain (Dataset 3, i.e., Dataset 1 in addition to reliable values extracted from Dataset 2, excluding low-quality data for problematic compounds), includes only consistent Log BMF_L_ values.

Several structural features generally related to the presence of heteroatoms, aromatic rings, chlorine atoms, as well as molecular polarizability and dimension have been highlighted as relevant for modelling Log BMF_L._

The models proposed in this study had comparable and good external predictive performances; however, Equation (4) had a larger applicability domain. They were both based on simple MLR equations that could be easily applied for the prediction of dietary BMF_L_ in fish, and support bioaccumulation assessment procedures at the regulatory level.

To ease the application and dissemination of these QSARs, they are both included with technical documentation (i.e., QMRF Reports) in QSAR-ME Profiler software for QSAR predictions, available from https://dunant.dista.uninsubria.it/qsar/ (accessed on 20 February 2023).

## Figures and Tables

**Table 1:** Averages of RMSE_test_ and MAE_test_ resulting from five-fold cross-validation of model based on Dataset 1.

Model	RMSE_test_	MAE_test_
k fold_a	0.85	0.61
k fold_b	1.06	0.78
k fold_c	0.70	0.55
k fold_d	0.78	0.52
k fold_e	0.58	0.48
Average	0.80	0.59

**Table 2:** List of the molecular descriptors included in Equation (1) and short description.

Molecular Descriptor	Description
AATS5i	Average Broto–Moreau autocorrelation—lag 5/weighted by first ionization potential
BCUTw-1l	N high lowest atom weighted BCUTS
PubchemFP257	≥2 aromatic rings
C3SP2	Number of doubly bounded carbons linked to 3 other carbons
MATS1i	Moran autocorrelation—lag 1/weighted by first ionization potential
GATS5m	Geary autocorrelation—lag 5/weighted by mass
GGI5	Topological charge index of order 5

**Table 3:** Averages of RMSE_test_ and MAE_test_ resulting from five-fold cross-validation based on Dataset 3.

Model	RMSE_test_	MAE_test_
k fold_a	0.53	0.38
k fold_b	0.73	0.47
k fold_c	0.57	0.40
k fold_d	0.69	0.45
k fold_e	0.72	0.44
Average	0.65	0.43

**Table 4:** List of the molecular descriptors included in Equation (3) and short description.

Molecular Descriptor	Description
PubchemFP503	Cl-C:C-[#1] (where “-” matches a single, “#” matches a triple bond and “:” denotes bond aromaticity)
SubFPC295	Counts of C–O, N or S bond
R_TpiPCTPC	Ratio of total conventional bond order (up to order 10) with total path count (up to order 10)
MLFER_S	Combined dipolarity/polarizability
maxHother	Maximum atom-type H E-State: H on aaCH, dCH2 or dsCH
GGI5	Topological charge index of order 5
VE3_Dt	Logarithmic coefficient sum of the last eigenvector from detour matrix

**Table 5:** Comparison of the statistical results of the QSAR models found in the literature with the models proposed in this study for the prediction of dietary Log BMF in fish. MLR: multiple linear regression, ANN: artificial neural network, GA: genetic algorithm, wNNR: weighted nearest-neighbour regression.

Authors	Method	Var.	Training					Prediction			Cross-Validation
			Response Range	N°	R^2^	RMSE	SE	N°	R^2^	RMSE	RMSE
Fatemi and Baher, 2009 [18]	(Stepwise-MLR) MLR	5	−0.13, 2.49	35	0.77 (R = 0.88)		0.24	7	0.50 (R = 0.71)		0.25
	(Stepwise-MLR)-ANN				0.98 (R = 0.99)		0.03		0.72 (R = 0.85)		0.11
	GA-MLR	4			0.72 (R = 0.85)		0.28		0.87 (R = 0.93)		0.27
	GA-ANN				1.0 (R = 1.0)		0.03		0.83 (R = 0.91)		0.08
Grisoni et al., 2019 [9]	wNNR	4	−4.50, 1.10	160	0.76	0.52		54	0.75	0.54	0.52
	MLR	7			0.75	0.53			0.71	0.57	0.55
	Consensus				0.81	0.47			0.82	0.45	0.49
Dataset 1 Equation (1)	GA-MLR	7	−2.30, 0.93	115	0.79	0.41		37	0.68	0.49	0.80
Dataset 3 Equation (3)			−4.49, 1.03	194	0.85	0.43		64	0.73	0.58	0.65

## Data Availability

Data used to generate the models proposed in this work are available as Supplementary material. Original raw data are available in J.A. Arnot and C.L. Quinn, “Development and evaluation of a database of dietary bioaccumulation test data for organic chemicals in fish,” *Environ. Sci. Technol.*, vol. 49, no. 8, pp. 4783–4796, 2015.

## Supplementary Materials

The following supporting information can be downloaded at: https://www.mdpi.com/article/10.3390/toxics11030209/s1. Table S1: Dataset 1: based on good quality data (high and medium). Chemicals are reported with CAS numbers, names, splitting category (Training or Prediction set), experimental and predicted Log BMFL, standardized residuals, HAT values and values of the molecular descriptors included in the model. Table S2: Dataset 2: Dataset based only on low quality data with experimental Log BMFL (low) and predicted Log BMFL (by Equation (2)). Table S3: Dataset 3: Consistent dataset based on high quality data (Dataset 1) and verified low quality data from Dataset 2. Chemicals are reported with CAS numbers, names, splitting category (Training or Prediction set), experimental and predicted Log BMFL, standardized residuals, HAT values and values of the molecular descriptors included in the model. Table S4: List of the excluded molecules. Table S5: List of the molecules outside the Applicability Domain (AD) of equations 1 and 3. with CAS numbers, SMILES, Structures, chemicals common names, and information on the AD.

## References

[1] European Chemicals Agency (2017). Guidance on Information Requirements and Chemical Safety Assessment: Chapter R.7c: Endpoint Specific Guidance.

[2] OECD (2012). Test No 305 Bioaccumulation in Fish: Aqueous and Dietary Exposure. Test No 305 Bioaccumulation Fish Aqueous Dietary Exposure.

[3] Gobas F.A.P.C., Lee Y.S., Arnot J.A. (2021). Normalizing the Biomagnification Factor. Environ. Toxicol. Chem..

[4] Hashizume N., Inoue Y., Suzuki Y., Murakami H., Sumi S., Ishibashi T., Yoshida T. (2018). Comparison of laboratory-derived biomagnification factors for hexachlorobenzene in common carp conducted under 9 test conditions. Environ. Toxicol. Chem..

[5] Gobas F.A.P.C., De Wolf W., Burkhard L.P., Verbruggen E., Plotzke K. (2009). Revisiting bioaccumulation criteria for POPs and PBT assessments. Integr. Environ. Assess. Manag..

[6] Burkhard L.P., Arnot J.A., Embry M.R., Farley K.J., Hoke R.A., Kitano M., Leslie H.A., Lotufo G.R., Parkerton T., Sappington K.G. (2012). Comparing laboratory and field measured bioaccumulation endpoints. Integr. Environ. Assess. Manag..

[7] Burkhard L.P., Cowan-Ellsberry C., Embry M.R., Hoke R.A., Kidd K.A. (2012). Bioaccumulation data from laboratory and field studies: Are they comparable?. Integr. Environ. Assess. Manag..

[8] Arnot J.A., Quinn C.L. (2015). Development and evaluation of a database of dietary bioaccumulation test data for organic chemicals in fish. Environ. Sci. Technol..

[9] Grisoni F., Consonni V., Vighi M. (2019). Acceptable-by-design QSARs to predict the dietary biomagnification of organic chemicals in fish. Integr. Environ. Assess. Manag..

[10] Grisoni F., Consonni V., Vighi M. (2018). Detecting the bioaccumulation patterns of chemicals through data-driven approaches. Chemosphere.

[11] Arnot J.A., Toose L., Armitage J.M., Embry M., Sangion A., Hughes L. (2022). A weight of evidence approach for bioaccumulation assessment. Integr. Environ. Assess. Manag..

[12] Franklin J. (2016). How reliable are field-derived biomagnification factors and trophic magnification factors as indicators of bioaccumulation potential? Conclusions from a case study on per- and polyfluoroalkyl substances. Integr. Environ. Assess. Manag..

[13] ECHA (2017). Chapter R.11: PBT/vPvB assessment. Guidance on Information Requirements and Chemical Safety Assessment.

[14] Gramatica P., Papa E. (2003). QSAR modeling of bioconcentration factor by theoretical molecular descriptors. QSAR Comb. Sci..

[15] Zhao C., Boriani E., Chana A., Roncaglioni A., Benfenati E. (2008). A new hybrid system of QSAR models for predicting bioconcentration factors (BCF). Chemosphere.

[16] Grisoni F., Consonni V., Vighi M., Villa S., Todeschini R. (2016). Expert QSAR system for predicting the bioconcentration factor under the REACH regulation. Environ. Res..

[17] Ivanciuc T., Ivanciuc O., Klein D.J. (2006). Modeling the bioconcentration factors and bioaccumulation factors of polychlorinated biphenyls with posetic quantitative super-structure/activity relationships (QSSAR). Mol. Divers..

[18] Fatemi M.H., Baher E. (2009). A novel quantitative structure-activity relationship model for prediction of biomagnification factor of some organochlorine pollutants. Mol. Divers..

[19] Chirico N., Bertato L., Papa E. (2022). QSAR Multiple Endpoint Profiler (QSAR-ME Profiler). http://dunant.dista.uninsubria.it/qsar/.

[20] NCI/CADD Group (2013). Chemical Identifier Resolver. https://cactus.nci.nih.gov.

[21] Berthold Michael R., Cebron N., Dill F. (2007). KNIME: The Konstanz Information Miner.

[22] O’Boyle N.M., Banck M., James C.A., Morley C., Vandermeersch T., Hutchison G.R. (2011). Open Babel: An open chemical toolbox. J. Cheminform..

[23] Yap C.W. (2010). PaDEL-descriptor: A software to calculate molecular descriptors and fingerprints. J. Comput. Chem..

[24] Gramatica P., Chirico N., Papa E., Cassani S., Kovarich S. (2013). QSARINS: A new software for the development, analysis, and validation of QSAR MLR models. J. Comput. Chem..

[25] Papa E., Kovarich S., Gramatica P. (2009). Development, Validation and Inspection of the Applicability Domain of QSPR Models for Physicochemical Properties of Polybrominated Diphenyl Ethers. QSAR Comb. Sci..

[26] Papa E., Villa F., Gramatica P. (2005). Statistically Validated QSARs, Based on Theoretical Descriptors, for Modeling Aquatic Toxicity of Organic Chemicals in Pimephales promelas (Fathead Minnow). J. Chem. Inf. Model..

[27] Leardi R., Boggia R., Terrile M. (1992). Genetic algorithms as a strategy for feature selection. J. Chemom..

[28] Tropsha A., Gramatica P., Gombar V.K. (2003). The Importance of Being Earnest: Validation is the absolute essential for successful application and interpretation of QSPR models. QSAR Comb. Sci..

[29] Pearlman R.S., Smith K.M. (1998). Novel software tools for chemical diversity. Perspect. Drug Discov. Des..

[30] Todeschini R., Consonni V. (2009). Molecular Descriptors for Chemoinformatics.

[31] Bertato L., Taboureau O., Chirico N., Papa E. (2022). Classification-based QSARs for predicting dietary biomagnification in fish. SAR QSAR Environ. Res..

[32] Lavado G.J., Baderna D., Gadaleta D., Ultre M., Roy K., Benfenati E. (2021). Ecotoxicological QSAR modeling of the acute toxicity of organic compounds to the freshwater crustacean Thamnocephalus platyurus. Chemosphere.

[33] Galvez J., Garcia R., Salabert M.T., Soler R. (1994). Charge Indexes. New Topological Descriptors. J. Chem. Inf. Comput. Sci..

[34] Bertato L., Chirico N., Papa E. (2022). Predicting the Bioconcentration Factor in Fish from Molecular Structures. Toxics.

[35] Doucette W.J., Shunthirasingham C., Dettenmaier E.M., Zaleski R.T., Fantke P., Arnot J.A. (2018). A review of measured bioaccumulation data on terrestrial plants for organic chemicals: Metrics, variability, and the need for standardized measurement protocols. Environ. Toxicol. Chem..

[36] Arnot J.A., Gobas F.A.P.C. (2006). A review of bioconcentration factor (BCF) and bioaccumulation factor (BAF) assessments for organic chemicals in aquatic organisms. Environ. Rev..

[37] Arnot J. (2021). EAS-E Suite—Exposure and Safety Estimation Suite. https://arnotresearch.com/eas-e-suite/.

[38] OECD (2004). Principles for the Validation, for Regulatory Purposes, of (Quantitative) Structure-Activity Relationship Models. https://www.oecd.org/chemicalsafety/risk-assessment/37849783.pdf.

