# Supplementary material for: QSAR Models for the Prediction of Dietary Biomagnification Factor in Fish
Source: Toxics. 2023 Feb 23;11(3):209. doi: 10.3390/toxics11030209 (PMC10054725; doi:10.3390/toxics11030209)
Supplement: Supplementary file 1 [file toxics-11-00209-s001.zip › toxics-2213758-supplementary.pdf]

# QSAR models for the prediction of dietary biomagnification factor in fish

Linda Bertato, Nicola Chirico and Ester Papa*

Department of Theoretical and Applied Sciences, University of Insubria, Varese, Italy

Supplementary materials

**Table S1** – Dataset 1: based on good quality data (high and medium). Chemicals are reported with CAS numbers, names, splitting category (Training or Prediction set), experimental and predicted Log $BMF_L$, standardized residuals, HAT values and values of the molecular descriptors included in the model.

**Table S2** – Dataset 2: Dataset based only on low quality data with experimental Log $BMF_L$ (low) and predicted Log $BMF_L$ (by Equation 2).

**Table S3** – Dataset 3: Consistent dataset based on high quality data (Dataset 1) and verified low quality data from Dataset 2. Chemicals are reported with CAS numbers, names, splitting category (Training or Prediction set), experimental and predicted Log $BMF_L$, standardized residuals, HAT values and values of the molecular descriptors included in the model.

**Table S4 -** List of the excluded molecules.

**Table S5 –** List of the molecules outside the Applicability Domain (AD) of equations 1 and 3. with CAS numbers, SMILES, Structures, chemicals common names, and information on the AD.

**Table S1 –** Dataset 1: based on good quality data (high and medium). Chemicals are reported with CAS numbers, names, splitting category (Training or Prediction set), experimental and predicted Log $BMF_L$, standardized residuals, HAT values and values of the molecular descriptors included in the model.

| **CAS** | **Chemical Name** | **Split Eq. 1** | **Exp Log $BMF_L$ highQuality** | **Pred Log $BMF_L$** | **Pred. Mod. Eq.Res.** | **Std.Pred. Mod. Eq. Res.** | **HAT i/i (h*=0.209)** | **AATS5i** | **BCUTw-1l** | **Pubchem FP257** | **C3SP2** | **MATS1i** | **GATS5m** | **GGI5** |
|---|---|---|---|---|---|---|---|---|---|---|---|---|---|---|
| **10394-57-7** | 9-n butylphenanthrene | Training | -1.854 | -1.174 | 0.680 | 1.610 | 0.022 | 152.312 | 11.890 | 1 | 5 | 0.053 | 1.095 | 0.389 |
| **104-72-3** | Decyl benzene | Prediction | -0.745 | -0.776 | -0.031 | -0.073 | 0.026 | 163.684 | 11.890 | 0 | 1 | 0.000 | 0.935 | 0.090 |
| **107534-96-3** | Tebuconazole | Training | -1.921 | -1.923 | -0.002 | -0.005 | 0.136 | 169.903 | 11.890 | 1 | 1 | -0.078 | 0.494 | 0.893 |
| **111-84-2** | n-nonane | Training | -0.893 | -1.195 | -0.302 | -0.722 | 0.038 | 167.659 | 11.890 | 0 | 0 | -0.079 | 0.919 | 0.056 |
| **112281-77-3** | Tetraconazole | Prediction | -2.046 | -1.411 | 0.634 | 1.635 | 0.173 | 170.118 | 11.988 | 1 | 1 | -0.118 | 1.037 | 0.847 |
| **112-40-3** | n-dodecane | Prediction | -0.878 | -1.056 | -0.179 | -0.425 | 0.031 | 166.549 | 11.890 | 0 | 0 | -0.059 | 0.946 | 0.056 |
| **112-41-4** | 1-dodecene | Training | -0.941 | -0.996 | -0.055 | -0.131 | 0.031 | 165.927 | 11.890 | 0 | 0 | -0.057 | 0.933 | 0.056 |
| **118-74-1** | Hexachlorobenzene | Training | 0.306 | 0.549 | 0.243 | 0.678 | 0.293 | 168.159 | 11.999 | 0 | 0 | 0.000 | 0.000 | 0.000 |
| **118-82-1** | Binox M | Prediction | 0.155 | -1.265 | -1.420 | -4.417 | 0.433 | 157.787 | 11.890 | 1 | 6 | 0.064 | 1.427 | 2.542 |
| **120068-37-3** | Fipronil | Training | -1.699 | -1.697 | 0.002 | 0.007 | 0.412 | 180.878 | 11.999 | 1 | 1 | -0.016 | 1.159 | 1.567 |
| **120-12-7** | Anthracene | Training | -1.875 | -1.651 | 0.224 | 0.538 | 0.049 | 155.231 | 11.850 | 1 | 4 | 0.055 | 0.962 | 0.111 |
| **123-48-8** | 2,2,4,4,6,6 pentamethyl-3-heptene | Prediction | -1.284 | -0.390 | 0.894 | 2.446 | 0.267 | 150.833 | 11.890 | 0 | 1 | -0.057 | 1.997 | 0.500 |
| **125116-23-6** | Metconazole | Training | -1.721 | -1.726 | -0.005 | -0.012 | 0.126 | 169.212 | 11.890 | 1 | 1 | -0.034 | 0.467 | 0.858 |
| **126690-66-2** | 2,4,6 Trimethyl-3-heptene | Prediction | -1.041 | -0.769 | 0.272 | 0.688 | 0.143 | 156.295 | 11.890 | 0 | 1 | -0.069 | 1.679 | 0.222 |
| **13029-08-8** | 2,2'-Dichlorophenyl | Prediction | 0.663 | 0.097 | -0.566 | -1.369 | 0.062 | 152.071 | 11.999 | 1 | 2 | 0.035 | 1.340 | 0.194 |
| **13150-81-7** | 2,6-Dimethyldecane | Training | -1.319 | -0.993 | 0.326 | 0.774 | 0.028 | 166.327 | 11.890 | 0 | 0 | -0.059 | 0.951 | 0.222 |

| | | | | | | | | | | | | | | |
|---|---|---|---|---|---|---|---|---|---|---|---|---|---|---|
| **13151-34-3** | 3-methyl decane | Prediction | -1.699 | -1.179 | 0.520 | 1.238 | 0.033 | 168.836 | 11.890 | 0 | 0 | -0.064 | 0.835 | 0.111 |
| **13475-82-6** | 2,2,4,6,6-pentamethylheptane | Training | -0.728 | -0.608 | 0.120 | 0.304 | 0.143 | 157.726 | 11.890 | 0 | 0 | -0.059 | 1.652 | 0.500 |
| **14233-37-5** | 9,10-Anthracenedione, 1,4-bis[(1-methylethyl)amino]- | Training | -0.500 | -0.857 | -0.357 | -0.883 | 0.103 | 152.222 | 11.890 | 1 | 4 | 0.017 | 1.124 | 1.389 |
| **15254-25-8** | 2,3,6,7-Tetramethylanthracene | Training | -1.757 | -2.096 | -0.339 | -0.838 | 0.102 | 160.517 | 11.890 | 1 | 8 | 0.053 | 0.819 | 0.444 |
| **15968-05-5** | 2,2',6,6'-Tetrachlorobiphenyl | Training | 0.623 | 0.420 | -0.203 | -0.488 | 0.046 | 149.757 | 11.999 | 1 | 2 | 0.035 | 1.106 | 0.250 |
| **1610-22-6** | 1,2,3,4,4a,7,8,9,10,11,12,12a-dodecahydrochrysene | Training | -0.770 | -0.496 | 0.273 | 0.663 | 0.068 | 159.450 | 11.900 | 0 | 4 | 0.089 | 1.072 | 0.361 |
| **16435-49-7** | 2-methyl-1-dodecene | Prediction | -1.200 | -1.102 | 0.099 | 0.234 | 0.029 | 165.756 | 11.890 | 0 | 1 | -0.053 | 0.940 | 0.111 |
| **16606-02-3** | 2,4',5-Trichlorobiphenyl | Training | 0.525 | 0.404 | -0.121 | -0.291 | 0.049 | 152.367 | 11.999 | 1 | 2 | 0.035 | 0.829 | 0.389 |
| **1678-98-4** | iso-butyl cyclohexane | Training | -0.652 | -0.695 | -0.044 | -0.104 | 0.024 | 165.164 | 11.900 | 0 | 0 | 0.000 | 1.036 | 0.118 |
| **16958-92-2** | Diisotridecyl adipate | Training | -1.959 | -1.017 | 0.942 | 2.251 | 0.040 | 165.093 | 11.890 | 0 | 0 | -0.085 | 1.023 | 0.222 |
| **1705-85-7** | 6-methylchyrsene | Training | -1.398 | -1.251 | 0.148 | 0.353 | 0.039 | 150.424 | 11.890 | 1 | 7 | 0.061 | 1.148 | 0.618 |
| **1706-50-9** | 1,1':3',1"Tercyclohexane | Training | -0.357 | -0.223 | 0.134 | 0.323 | 0.062 | 163.993 | 11.900 | 0 | 0 | 0.068 | 0.937 | 0.500 |
| **17088-22-1** | 1-ethylpyrene | Training | -1.699 | -1.424 | 0.275 | 0.657 | 0.040 | 152.141 | 11.890 | 1 | 7 | 0.067 | 1.162 | 0.458 |
| **17301-23-4** | 2,6-Dimethyl undecane | Training | -1.027 | -0.950 | 0.077 | 0.183 | 0.028 | 166.145 | 11.890 | 0 | 0 | -0.054 | 0.956 | 0.278 |
| **17312-44-6** | 2,3 Dimethyldecane | Prediction | -1.585 | -1.196 | 0.389 | 0.928 | 0.034 | 169.888 | 11.890 | 0 | 0 | -0.059 | 0.774 | 0.167 |
| **1732-13-4** | 1,2,3,6,7,8 Hexahydropyrene | Training | -1.252 | -1.471 | -0.219 | -0.527 | 0.051 | 154.831 | 11.900 | 1 | 6 | 0.086 | 1.229 | 0.111 |
| **1795-15-9** | n - Octyl Cyclohexane | Training | -1.222 | -0.752 | 0.470 | 1.114 | 0.025 | 166.787 | 11.900 | 0 | 0 | 0.000 | 0.887 | 0.090 |

| | | | | | | | | | | | | | |
|---|---|---|---|---|---|---|---|---|---|---|---|---|---|
| **18094-01-4** | 2 methyl 1-Tridecene | Prediction | -0.921 | -1.081 | -0.160 | -0.381 | 0.028 | 165.618 | 11.890 | 0 | 1 | -0.049 | 0.945 | 0.111 |
| **1836-75-5** | 2,4-Dichloro-1-(4-nitrophenoxy) benzene | Training | -0.821 | -0.485 | 0.336 | 0.910 | 0.254 | 154.242 | 11.850 | 1 | 0 | 0.168 | 0.854 | 0.417 |
| **1839-63-0** | 1,3,5-Trimethyl cyclohexane | Training | -0.456 | -0.954 | -0.498 | -1.192 | 0.044 | 166.510 | 11.900 | 0 | 0 | 0.000 | 1.254 | 0.000 |
| **18516-37-5** | 2-methyl-1-undecene | Training | -1.456 | -1.126 | 0.330 | 0.785 | 0.031 | 165.927 | 11.890 | 0 | 1 | -0.057 | 0.933 | 0.111 |
| **1889-67-4** | Benzene, 1,1'-(1,1,2,2-tetramethyl-1,2-ethaneDiyl)bis- | Training | -0.777 | -1.424 | -0.646 | -1.553 | 0.050 | 162.220 | 11.890 | 1 | 2 | 0.030 | 0.911 | 0.625 |
| **191-24-2** | Benzo(ghi)perylene | Training | -1.495 | -1.618 | -0.123 | -0.305 | 0.111 | 145.804 | 11.850 | 1 | 10 | 0.070 | 1.085 | 0.736 |
| **193-39-5** | Indeno 1,2,3-cd pyrene | Training | -1.538 | -1.634 | -0.096 | -0.239 | 0.112 | 146.060 | 11.850 | 1 | 10 | 0.070 | 1.124 | 0.820 |
| **194-69-4** | Benzo(c)chrysene | Training | -1.301 | -1.600 | -0.299 | -0.723 | 0.061 | 148.346 | 11.850 | 1 | 8 | 0.064 | 1.079 | 0.542 |
| **19780-74-6** | 5-ethyl-1-nonene | Training | -0.461 | -0.984 | -0.523 | -1.244 | 0.030 | 166.134 | 11.890 | 0 | 0 | -0.063 | 0.909 | 0.167 |
| **20279-21-4** | 1,2,3,10b-Tetrahydrofluoranthene | Training | -1.638 | -1.252 | 0.386 | 0.920 | 0.033 | 154.143 | 11.900 | 1 | 5 | 0.080 | 1.217 | 0.219 |
| **204256-07-5** | 2,3 Dimethy-5(4methylpentyl) naphthalene | Training | -1.367 | -1.748 | -0.381 | -0.909 | 0.034 | 160.151 | 11.890 | 1 | 5 | 0.031 | 0.951 | 0.514 |
| **2050-68-2** | 4,4'-Dichlorobiphenyl | Training | 0.519 | 0.460 | -0.058 | -0.144 | 0.110 | 153.991 | 11.999 | 1 | 2 | 0.035 | 0.471 | 0.361 |
| **2051-30-1** | 2,6-Dimethyl octane | Prediction | -1.854 | -1.201 | 0.652 | 1.555 | 0.034 | 169.400 | 11.890 | 0 | 0 | -0.071 | 0.814 | 0.222 |
| **206-44-0** | Fluoranthene | Training | -1.553 | -1.585 | -0.032 | -0.077 | 0.057 | 149.167 | 11.850 | 1 | 6 | 0.065 | 1.303 | 0.219 |
| **207-08-9** | Benzo(k)fluoranthene | Prediction | -1.979 | -1.614 | 0.365 | 0.881 | 0.060 | 148.762 | 11.850 | 1 | 8 | 0.067 | 1.091 | 0.594 |
| **214-17-5** | Benzo[b]chrysene | Training | -1.745 | -1.721 | 0.023 | 0.056 | 0.058 | 150.131 | 11.850 | 1 | 8 | 0.064 | 1.034 | 0.528 |
| **217-59-4** | Triphenylene | Training | -1.658 | -1.311 | 0.346 | 0.835 | 0.056 | 146.996 | 11.850 | 1 | 6 | 0.061 | 1.175 | 0.375 |
| **218-01-9** | Chrysene | Prediction | -1.538 | -1.598 | -0.061 | -0.145 | 0.041 | 150.909 | 11.850 | 1 | 6 | 0.061 | 1.125 | 0.361 |
| **2189-60-8** | n-octyl benzene | Prediction | -1.648 | -0.759 | 0.888 | 2.109 | 0.026 | 163.529 | 11.890 | 0 | 1 | 0.000 | 0.927 | 0.090 |
| **22907-72-8** | 1,4 Diisopropyl cyclohexane | Prediction | -0.479 | -1.009 | -0.530 | -1.262 | 0.032 | 170.097 | 11.890 | 0 | 0 | 0.000 | 0.844 | 0.361 |

| | | | | | | | | | | | | | | |
|---|---|---|---|---|---|---|---|---|---|---|---|---|---|---|
| **23342-25-8** | 2,2,5,7-Tetramethyltetraline | Prediction | -1.301 | -0.796 | 0.505 | 1.220 | 0.060 | 159.824 | 11.890 | 0 | 4 | 0.041 | 1.207 | 0.535 |
| **243-17-4** | Benzo(b)fluorene | Training | -1.602 | -1.197 | 0.405 | 0.963 | 0.028 | 153.527 | 11.900 | 1 | 6 | 0.066 | 0.978 | 0.445 |
| **2435-85-0** | Hexadecahydropyrene | Training | 0.009 | -0.295 | -0.304 | -0.738 | 0.071 | 164.555 | 11.900 | 0 | 0 | 0.108 | 1.010 | 0.111 |
| **2437-56-1** | 1-Tridecene | Training | -0.963 | -0.972 | -0.009 | -0.022 | 0.030 | 165.756 | 11.890 | 0 | 0 | -0.053 | 0.940 | 0.056 |
| **2541-69-7** | 7-Methylbenz(a)anthracene | Training | -2.097 | -1.364 | 0.733 | 1.750 | 0.038 | 151.919 | 11.890 | 1 | 7 | 0.061 | 1.121 | 0.583 |
| **25569-80-6** | 2,3'-Dichlorobiphenyl | Training | 0.267 | 0.099 | -0.169 | -0.406 | 0.055 | 152.564 | 11.999 | 1 | 2 | 0.035 | 1.340 | 0.333 |
| **26186-00-5** | 1-Heptadecyne | Training | -0.086 | -0.863 | -0.777 | -1.847 | 0.029 | 164.854 | 11.890 | 0 | 0 | -0.039 | 0.948 | 0.056 |
| **2732-58-3** | 6-ethylchyrsene | Prediction | -1.495 | -1.200 | 0.295 | 0.705 | 0.041 | 150.325 | 11.890 | 1 | 7 | 0.062 | 1.119 | 0.708 |
| **2974-92-7** | 3,4-Dichlorobiphenyl | Training | 0.477 | 0.362 | -0.115 | -0.277 | 0.061 | 153.530 | 11.999 | 1 | 2 | 0.035 | 0.722 | 0.361 |
| **2980-71-4** | 2-methyl-1-nonene | Prediction | -1.367 | -1.194 | 0.173 | 0.413 | 0.035 | 166.432 | 11.890 | 0 | 1 | -0.069 | 0.916 | 0.111 |
| **3074-71-3** | 2,3-Dimethylheptane | Prediction | -0.792 | -1.397 | -0.605 | -1.454 | 0.050 | 171.973 | 11.890 | 0 | 0 | -0.079 | 0.689 | 0.111 |
| **319-84-6** | (1 alpha,2 alpha,3 beta,4 alpha,5 beta,6 beta)1,2,3,4,5,6-Hexachlorocyclohexane | Training | -1.516 | -0.834 | 0.682 | 1.789 | 0.203 | 176.439 | 11.999 | 0 | 0 | 0.000 | 1.358 | 0.000 |
| **32774-16-6** | 3,3',4,4',5,5'-Hexachlorobiphenyl | Training | 0.496 | 0.434 | -0.061 | -0.146 | 0.036 | 151.311 | 11.999 | 1 | 2 | 0.035 | 1.031 | 0.583 |
| **33025-41-1** | 2,3,4,4'-Tetrachlorobiphenyl | Training | 0.688 | 0.353 | -0.335 | -0.799 | 0.035 | 152.146 | 11.999 | 1 | 2 | 0.035 | 1.015 | 0.500 |
| **3321-50-4** | Cyclohexane, 1,1'-(1,2-EthaneDiyl)Bis- | Training | -1.301 | -0.581 | 0.721 | 1.726 | 0.044 | 167.485 | 11.900 | 0 | 0 | 0.045 | 0.779 | 0.181 |
| **33460-02-5** | 3 Phenyl bicyclohexyl | Training | -1.046 | -1.102 | -0.056 | -0.133 | 0.029 | 165.756 | 11.890 | 0 | 1 | -0.053 | 0.940 | 0.111 |
| **34883-43-7** | 2,4'-Dichlorobiphenyl | Training | 0.574 | 0.180 | -0.394 | -0.942 | 0.041 | 153.025 | 11.999 | 1 | 2 | 0.035 | 1.090 | 0.278 |

| | | | | | | | | | | | | | | |
|---|---|---|---|---|---|---|---|---|---|---|---|---|---|---|
| **35065-27-1** | 2,2',4,4',5,5'-Hexachlorobiphenyl | Training | 0.614 | 0.661 | 0.047 | 0.114 | 0.058 | 150.336 | 11.999 | 1 | 2 | 0.035 | 0.813 | 0.694 |
| **37680-65-2** | 2,2',5-Trichlorobiphenyl | Training | 0.580 | 0.364 | -0.216 | -0.516 | 0.039 | 151.413 | 11.999 | 1 | 2 | 0.035 | 1.029 | 0.361 |
| **38380-01-7** | 2,2',4,4',5-Pentachlorobiphenyl | Prediction | 0.868 | 0.535 | -0.333 | -0.799 | 0.045 | 150.995 | 11.999 | 1 | 2 | 0.035 | 0.891 | 0.583 |
| **38444-73-4** | 2,2',6-Trichlorobiphenyl | Prediction | 0.568 | 0.205 | -0.363 | -0.876 | 0.058 | 150.908 | 11.999 | 1 | 2 | 0.035 | 1.323 | 0.222 |
| **38444-77-8** | 2,4',6-Trichlorobiphenyl | Training | 0.580 | 0.105 | -0.475 | -1.151 | 0.066 | 151.851 | 11.999 | 1 | 2 | 0.035 | 1.417 | 0.306 |
| **38444-78-9** | 2,2',3-Trichlorobiphenyl | Prediction | 0.477 | 0.207 | -0.270 | -0.650 | 0.051 | 151.401 | 11.999 | 1 | 2 | 0.035 | 1.323 | 0.361 |
| **38444-85-8** | 2,3,4'-Trichlorobiphenyl | Prediction | 0.352 | 0.247 | -0.105 | -0.251 | 0.037 | 152.355 | 11.999 | 1 | 2 | 0.035 | 1.123 | 0.389 |
| **38444-86-9** | 2,3',4'-Trichlorobiphenyl | Prediction | 0.519 | 0.263 | -0.255 | -0.609 | 0.036 | 152.355 | 11.999 | 1 | 2 | 0.035 | 1.123 | 0.444 |
| **3891-98-3** | 2,6,10-Trimethyl dodecane | Training | -0.002 | -0.946 | -0.944 | -2.244 | 0.028 | 167.226 | 11.890 | 0 | 0 | -0.046 | 0.894 | 0.389 |
| **40458-98-8** | 2,7-Diisopropyl naphthalene | Training | -1.438 | -1.594 | -0.156 | -0.372 | 0.031 | 160.496 | 11.890 | 1 | 4 | 0.034 | 0.940 | 0.583 |
| **41411-64-7** | 2,3,3',4,4',5,6-Heptachlorobiphenyl | Training | 0.894 | 0.527 | -0.367 | -0.878 | 0.044 | 149.655 | 11.999 | 1 | 2 | 0.035 | 1.194 | 0.750 |
| **41464-39-5** | 2,2',3,5'-Tetrachlorobiphenyl | Training | 0.789 | 0.407 | -0.382 | -0.912 | 0.037 | 150.743 | 11.999 | 1 | 2 | 0.035 | 1.106 | 0.472 |
| **41464-49-7** | 2,3,3',5'-Tetrachlorobiphenyl | Prediction | 0.238 | 0.241 | 0.003 | 0.007 | 0.052 | 151.224 | 11.999 | 1 | 2 | 0.035 | 1.360 | 0.500 |
| **4175-54-6** | 1,2,3,4-Tetrahydro-1,4-Dimethyl naphthalene (1,4-Dimethyl Tetralin) | Training | -0.801 | -0.831 | -0.029 | -0.070 | 0.031 | 163.515 | 11.890 | 0 | 2 | 0.046 | 1.079 | 0.125 |
| **4292-75-5** | n-hexyl cyclohexane | Training | -1.377 | -0.783 | 0.594 | 1.409 | 0.026 | 167.334 | 11.900 | 0 | 0 | 0.000 | 0.864 | 0.090 |
| **43121-43-3** | Triadimefon | Prediction | -1.921 | -2.023 | -0.102 | -0.266 | 0.188 | 167.398 | 11.890 | 1 | 0 | -0.183 | 0.697 | 0.871 |
| **4316-65-8** | 3,5,5' Trimethyl hexene | Training | -1.201 | -1.019 | 0.181 | 0.439 | 0.065 | 162.847 | 11.890 | 0 | 0 | -0.077 | 1.284 | 0.000 |

| | | | | | | | | | | | | | | |
|---|---|---|---|---|---|---|---|---|---|---|---|---|---|---|
| **438-22-2** | 5α(H)-androstane | Prediction | -0.172 | -0.245 | -0.073 | -0.178 | 0.090 | 165.899 | 11.900 | 0 | 0 | 0.094 | 0.935 | 0.667 |
| **4390-04-9** | 2,2,4,4,6,8,8-heptamethyl nonane | Prediction | 0.004 | -0.340 | -0.344 | -0.910 | 0.216 | 154.865 | 11.890 | 0 | 0 | -0.043 | 1.814 | 0.778 |
| **475-03-6** | 1,1,6 Trimethyl Tetralin | Training | -0.484 | -0.858 | -0.374 | -0.894 | 0.039 | 162.053 | 11.890 | 0 | 3 | 0.043 | 1.163 | 0.319 |
| **488-23-3** | 1,2,3,4 Tetramethyl benzene | Training | -1.292 | -1.774 | -0.481 | -1.189 | 0.100 | 171.512 | 11.890 | 0 | 4 | 0.000 | 0.777 | 0.000 |
| **4920-95-0** | 3,3',4,4' Tetramethyl 1,1'-biphenyl | Training | -2.046 | -2.069 | -0.023 | -0.057 | 0.080 | 164.109 | 11.890 | 1 | 6 | 0.032 | 0.708 | 0.528 |
| **496-10-6** | Bicyclo[4.3.0]nonane | Training | -1.102 | -1.056 | 0.047 | 0.114 | 0.079 | 174.319 | 11.900 | 0 | 0 | 0.068 | 0.694 | 0.000 |
| **50-32-8** | Benzo[a]pyrene | Training | -1.745 | -1.657 | 0.088 | 0.212 | 0.062 | 148.080 | 11.850 | 1 | 8 | 0.067 | 1.151 | 0.375 |
| **50876-32-9** | cis 1,1,3,5 Tetramethyl cyclohexane | Training | -0.265 | -0.801 | -0.536 | -1.310 | 0.082 | 162.838 | 11.900 | 0 | 0 | 0.000 | 1.510 | 0.000 |
| **51655-65-3** | 2-butyl-1-decene | Prediction | -0.975 | -1.037 | -0.063 | -0.149 | 0.029 | 164.953 | 11.890 | 0 | 1 | -0.049 | 0.961 | 0.111 |
| **52663-59-9** | 2,2',3,4-Tetrachlorobiphenyl | Training | 0.218 | 0.328 | 0.111 | 0.264 | 0.038 | 151.192 | 11.999 | 1 | 2 | 0.035 | 1.187 | 0.472 |
| **526-73-8** | 1,3,5-Trimethylbenzene | Training | -1.602 | -1.720 | -0.118 | -0.288 | 0.084 | 172.994 | 11.890 | 0 | 3 | 0.000 | 0.729 | 0.000 |
| **52886-35-8** | 3-methyl-1-hexyl cyclohexane | Training | -1.181 | -0.739 | 0.441 | 1.046 | 0.023 | 166.495 | 11.900 | 0 | 0 | 0.000 | 0.936 | 0.146 |
| **5325-97-3** | 1,2,3,4,5,6,7,8-octahydrophenanthrene | Training | -0.893 | -0.633 | 0.260 | 0.629 | 0.062 | 159.370 | 11.900 | 0 | 4 | 0.076 | 1.132 | 0.125 |
| **53-70-3** | Dibenzo[a,h]anthracene | Training | -2.155 | -1.721 | 0.434 | 1.046 | 0.058 | 150.131 | 11.850 | 1 | 8 | 0.064 | 1.034 | 0.528 |
| **541-02-6** | Decamethylcyclopentasiloxane | Training | 0.532 | 1.007 | 0.475 | 1.380 | 0.350 | 155.496 | 11.998 | 0 | 0 | -0.178 | 1.027 | 1.944 |
| **556-67-2** | Octamethylcyclotetrasiloxane | Training | 0.602 | 0.374 | -0.228 | -0.584 | 0.160 | 160.019 | 11.998 | 0 | 0 | -0.178 | 0.994 | 0.944 |
| **55702-45-9** | 2,3,6-Trichlorobiphenyl | Training | 0.470 | 0.191 | -0.279 | -0.672 | 0.054 | 151.401 | 11.999 | 1 | 2 | 0.035 | 1.323 | 0.306 |
| **55712-37-3** | 2,3',4-Trichlorobiphenyl | Training | 0.556 | 0.263 | -0.293 | -0.699 | 0.036 | 152.355 | 11.999 | 1 | 2 | 0.035 | 1.123 | 0.444 |
| **5617-41-4** | n-heptyl cyclohexane | Training | -1.699 | -0.766 | 0.933 | 2.214 | 0.026 | 167.033 | 11.900 | 0 | 0 | 0.000 | 0.877 | 0.090 |

| | | | | | | | | | | | | | | |
|---|---|---|---|---|---|---|---|---|---|---|---|---|---|---|
| **56-49-5** | 3-methylcholanthene | Training | -1.523 | -1.700 | -0.177 | -0.433 | 0.086 | 152.912 | 11.890 | 1 | 9 | 0.074 | 1.141 | 0.601 |
| **56558-16-8** | 2,2',4,6,6'-Pentachlorobiphenyl | Training | 0.393 | 0.500 | 0.107 | 0.257 | 0.043 | 149.548 | 11.999 | 1 | 2 | 0.035 | 1.048 | 0.361 |
| **5707-44-8** | 4-ethyl-1,1'-biphenyl | Prediction | -1.745 | -1.390 | 0.354 | 0.843 | 0.029 | 158.741 | 11.890 | 1 | 3 | 0.034 | 0.947 | 0.313 |
| **57465-28-8** | 3,3',4,4',5-Pentachlorobiphenyl | Prediction | 0.581 | 0.457 | -0.124 | -0.297 | 0.042 | 151.981 | 11.999 | 1 | 2 | 0.035 | 0.891 | 0.583 |
| **581-40-8** | 2,3-Dimethylnaphthalene | Training | -1.921 | -1.894 | 0.027 | 0.066 | 0.054 | 163.525 | 11.890 | 1 | 4 | 0.040 | 0.861 | 0.167 |
| **608-93-5** | Pentachlorobenzene | Prediction | -0.387 | -0.272 | 0.115 | 0.286 | 0.107 | 170.886 | 11.999 | 0 | 0 | 0.000 | 1.128 | 0.000 |
| **6117-97-1** | 4-methyl dodecane | Training | -1.114 | -1.019 | 0.095 | 0.225 | 0.029 | 166.090 | 11.890 | 0 | 0 | -0.054 | 1.001 | 0.111 |
| **62338-09-4** | 2,2,3-Trimethyl decane | Training | -1.181 | -1.200 | -0.019 | -0.046 | 0.036 | 170.617 | 11.890 | 0 | 0 | -0.054 | 0.732 | 0.222 |
| **629-50-5** | n-Tridecane | Training | -0.569 | -1.027 | -0.458 | -1.090 | 0.030 | 166.327 | 11.890 | 0 | 0 | -0.054 | 0.951 | 0.056 |
| **629-59-4** | n-Tetradecane | Training | 0.029 | -1.002 | -1.032 | -2.453 | 0.029 | 166.145 | 11.890 | 0 | 0 | -0.050 | 0.956 | 0.056 |
| **629-73-2** | 1-Hexadecene | Training | 0.183 | -0.920 | -1.103 | -2.621 | 0.029 | 165.408 | 11.890 | 0 | 0 | -0.043 | 0.953 | 0.056 |
| **66246-88-6** | 1H-1,2,4-Triazole, 1-[2-(2,4-Dichlorophenyl)pentyl]- | Training | -2.000 | -1.794 | 0.206 | 0.512 | 0.109 | 162.141 | 11.890 | 1 | 1 | -0.124 | 0.951 | 0.569 |
| **68194-04-7** | 2,2',4,6'-Tetrachlorobiphenyl | Training | -0.854 | 0.326 | 1.180 | 2.825 | 0.043 | 150.699 | 11.999 | 1 | 2 | 0.035 | 1.187 | 0.333 |
| **68194-05-8** | 2,2',3,4',6-Pentachlorobiphenyl | Training | 0.303 | 0.502 | 0.198 | 0.474 | 0.039 | 150.041 | 11.999 | 1 | 2 | 0.035 | 1.048 | 0.500 |
| **68194-15-0** | 2,2',3,4,5,6'-Hexachlorobiphenyl | Prediction | -0.523 | 0.565 | 1.088 | 2.608 | 0.045 | 149.371 | 11.999 | 1 | 2 | 0.035 | 1.180 | 0.778 |
| **68194-16-1** | 2,2',3,3',4,5,6-Heptachlorobiphenyl | Training | 0.009 | 0.647 | 0.638 | 1.531 | 0.047 | 148.712 | 11.999 | 1 | 2 | 0.035 | 1.125 | 0.778 |

| | | | | | | | | | | | | | |
|---|---|---|---|---|---|---|---|---|---|---|---|---|---|
| **6975-98-0** | 2-methyl decane | Training | -1.444 | -1.076 | 0.368 | 0.875 | 0.031 | 166.826 | 11.890 | 0 | 0 | -0.064 | 0.939 | 0.111 |
| **702-79-4** | 1,3-Dimethyladamantane | Training | -1.174 | -0.491 | 0.683 | 1.674 | 0.087 | 163.721 | 11.900 | 0 | 0 | 0.098 | 1.378 | 0.000 |
| **70356-09-1** | 1-(4-tert-butylphenyl)-3-(4-methoxyphenyl)propane-1,3-Dione | Training | -0.914 | -1.508 | -0.593 | -1.439 | 0.067 | 161.118 | 11.890 | 1 | 3 | -0.022 | 0.889 | 1.056 |
| **7045-71-8** | 2-methyl undecane | Training | -1.292 | -1.040 | 0.252 | 0.600 | 0.030 | 166.549 | 11.890 | 0 | 0 | -0.059 | 0.946 | 0.111 |
| **7116-96-3** | 4-pentyl-1,1'-Biphenyl | Prediction | -1.174 | -1.379 | -0.205 | -0.487 | 0.029 | 158.588 | 11.890 | 1 | 3 | 0.031 | 0.942 | 0.340 |
| **71608-00-9** | 1-octylpyrene | Prediction | -1.108 | -1.643 | -0.535 | -1.283 | 0.044 | 155.729 | 11.890 | 1 | 7 | 0.061 | 1.026 | 0.486 |
| **717-74-8** | Benzene, 1,3,5-Tris(1-methylethyl)- | Training | -0.398 | -0.492 | -0.094 | -0.233 | 0.097 | 159.698 | 11.890 | 0 | 3 | 0.000 | 0.969 | 1.042 |
| **72-43-5** | Methoxy chlor | Training | -0.969 | -0.703 | 0.266 | 0.668 | 0.126 | 158.028 | 11.996 | 1 | 2 | -0.092 | 1.415 | 0.653 |
| **73575-52-7** | 2,3',4,5'-Tetrachlorobiphenyl | Training | 0.218 | 0.330 | 0.112 | 0.268 | 0.038 | 151.685 | 11.999 | 1 | 2 | 0.035 | 1.187 | 0.611 |
| **74472-36-9** | 2,3,3',5,6-Pentachlorobiphenyl | Training | 0.477 | 0.423 | -0.054 | -0.130 | 0.040 | 150.072 | 11.999 | 1 | 2 | 0.035 | 1.205 | 0.528 |
| **74472-38-1** | 2,3,4,4',6-Pentachlorobiphenyl | Training | 0.857 | 0.396 | -0.461 | -1.100 | 0.036 | 150.983 | 11.999 | 1 | 2 | 0.035 | 1.122 | 0.528 |
| **74472-48-3** | 2,2',3,4,4',6,6'-Heptachlorobiphenyl | Training | 0.928 | 0.703 | -0.225 | -0.541 | 0.049 | 148.681 | 11.999 | 1 | 2 | 0.035 | 0.980 | 0.694 |
| **74487-85-7** | 2,2',3,4',5,6,6'-Heptachlorobiphenyl | Training | 0.884 | 0.807 | -0.076 | -0.184 | 0.061 | 148.231 | 11.999 | 1 | 2 | 0.035 | 0.912 | 0.806 |
| **781-17-9** | 4,5,9,10-Tetrahydropyrene | Training | -1.553 | -1.486 | 0.067 | 0.162 | 0.050 | 154.874 | 11.900 | 1 | 6 | 0.080 | 1.214 | 0.111 |
| **81-15-2** | Musk-xylene | Training | -0.210 | -0.392 | -0.182 | -0.600 | 0.495 | 169.015 | 11.890 | 0 | 3 | 0.278 | 1.353 | 1.618 |
| **821-95-4** | 1-undecene | Prediction | -1.409 | -1.026 | 0.383 | 0.912 | 0.033 | 166.145 | 11.890 | 0 | 0 | -0.063 | 0.926 | 0.056 |

| | | | | | | | | | | | | | | |
|---|---|---|---|---|---|---|---|---|---|---|---|---|---|---|
| **832-69-9** | 1-Methylphenanthrene | Training | -1.721 | -1.408 | 0.314 | 0.744 | 0.026 | 154.746 | 11.890 | 1 | 5 | 0.055 | 1.138 | 0.299 |
| **84-15-1** | o-Terphenyl | Training | -0.499 | -1.087 | -0.588 | -1.429 | 0.072 | 148.112 | 11.850 | 1 | 4 | 0.046 | 1.027 | 0.333 |
| **85-01-8** | Phenanthrene | Training | -1.122 | -1.616 | -0.494 | -1.188 | 0.053 | 153.631 | 11.850 | 1 | 4 | 0.055 | 1.139 | 0.125 |
| **872-05-9** | 1-decene | Training | -0.991 | -1.064 | -0.072 | -0.173 | 0.034 | 166.432 | 11.890 | 0 | 0 | -0.069 | 0.916 | 0.056 |
| **88671-89-0** | Myclobutanil | Training | -2.097 | -1.536 | 0.561 | 1.390 | 0.105 | 162.237 | 11.890 | 1 | 1 | -0.107 | 0.619 | 0.684 |
| **91-17-8** | t-decalin | Training | -0.068 | -0.897 | -0.830 | -2.004 | 0.060 | 171.594 | 11.900 | 0 | 0 | 0.062 | 0.764 | 0.000 |
| **91-20-3** | Naphthalene | Training | -2.301 | -2.034 | 0.267 | 0.656 | 0.090 | 162.291 | 11.850 | 1 | 2 | 0.042 | 1.043 | 0.000 |
| **91-57-6** | 2-Methylnaphthalene | Training | -2.000 | -1.588 | 0.412 | 0.988 | 0.045 | 159.982 | 11.890 | 1 | 3 | 0.041 | 1.047 | 0.083 |
| **92-06-8** | m-terphenyl | Training | -1.347 | -1.266 | 0.081 | 0.196 | 0.057 | 151.381 | 11.850 | 1 | 4 | 0.046 | 0.969 | 0.500 |
| **92-51-3** | Bicyclohexyl | Training | -0.770 | -0.542 | 0.227 | 0.543 | 0.039 | 166.327 | 11.900 | 0 | 0 | 0.052 | 0.897 | 0.139 |
| **933-12-0** | 3,5,5 Trimethyl cyclohexene | Training | -1.137 | -0.943 | 0.194 | 0.463 | 0.036 | 166.852 | 11.900 | 0 | 0 | 0.000 | 1.184 | 0.000 |
| **93-46-9** | N,N′-Di-2-naphthyl-p-phenyleneDiamine | Training | -1.096 | -1.512 | -0.416 | -0.998 | 0.047 | 155.106 | 11.850 | 1 | 4 | 0.074 | 0.966 | 0.361 |
| **94361-06-5** | Cyproconazole | Training | -1.959 | -1.405 | 0.554 | 1.354 | 0.081 | 164.796 | 11.900 | 1 | 1 | -0.037 | 0.639 | 0.771 |
| **98-51-1** | 1-tert butyl-4-methyl benzene | Training | -1.018 | -1.015 | 0.003 | 0.006 | 0.027 | 165.778 | 11.890 | 0 | 2 | 0.000 | 0.945 | 0.354 |

**Table S2** – Dataset 2: Dataset based only on low quality with experimental Log BMF$_L$ (low) and predicted Log BMF$_L$ (by Equation 2).

| CAS | Chemical Name | SMILES | Log_BMF$_L$ LowQuality | Log_BMF$_L$ Pred. by model eq. |
|---|---|---|---|---|
| **1024-57-3** | 2,3,4,5,6,7,7-Heptachloro-1a,1b,5,5a,6,6a,-Hexahydro-(2a alpha, 1b beta, 2 alpha, 5 alpha, 5a beta, 6 beta, 6a alpha-2,5-methano-2H-indeno[1,2-b]oxirene | ClC1C2OC2C2C1C1(Cl)C(=C(C2(C1(Cl)Cl)Cl)Cl)Cl | 0.415 | 0.319 |
| **10605-21-7** | Carbendazim | COC(=O)Nc1nc2c([nH]1)cccc2 | -3.745 | -1.807 |
| **117-81-7** | Di-2-Ethylhexyl phthalate | CCCCC(COC(=O)c1ccccc1C(=O)OCC(CCCC)CC)CC | -1.125 | -1.041 |
| **120068-36-2** | Fipronil sulfone | N#Cc1nn(c(c1S(=O)(=O)C(F)(F)F)N)c1c(Cl)cc(cc1Cl)C(F)(F)F | 0.777 | -1.418 |
| **129-00-0** | Pyrene | c1cc2ccc3c4c2c(c1)ccc4ccc3 | -2.301 | -1.771 |
| **131860-33-8** | Azoxystrobin | COC=C(c1ccccc1Oc1ncnc(c1)Oc1ccccc1C#N)C(=O)OC | -3.347 | -2.205 |
| **15299-99-7** | Napropamide | CCN(C(=O)C(Oc1cccc2c1cccc2)C)CC | -2.852 | -2.203 |
| **1746-01-6** | 2,3,7,8-Tetrachlorodibenzo[b,e][1,4]Dioxin | Clc1cc2Oc3cc(Cl)c(cc3Oc2cc1Cl)Cl | 0.103 | 0.612 |
| **2051-24-3** | Decachlorobiphenyl | Clc1c(c2c(Cl)c(Cl)c(c(c2Cl)Cl)Cl)c(Cl)c(c(c1Cl)Cl)Cl | 0.319 | 1.048 |
| **205646-11-3** | 1,2,9,10-Tetrachlorodecane | ClCC(CCCCCCC(CCl)Cl)Cl | -0.658 | 0.035 |
| **208465-21-8** | Mesosulfuron-methyl | COC(=O)c1ccc(cc1S(=O)(=O)NC(=O)Nc1nc(OC)cc(n1)OC)CNS(=O)(=O)C | -5.699 | -1.274 |
| **2136-99-4** | 2,2',3,3',5,5',6,6'-Octachlorobiphenyl | Clc1c(Cl)cc(c(c1c1c(Cl)c(Cl)cc(c1Cl)Cl)Cl)Cl | 0.667 | 1.026 |
| **221155-23-3** | 1,2,13,14-Tetrachlorotetradecane | ClCC(CCCCCCCCCCC(CCl)Cl)Cl | 0.100 | 0.194 |
| **2381-21-7** | 1-Methylpyrene | Cc1ccc2c3c1ccc1c3c(cc2)ccc1 | -2.301 | -1.489 |
| **2385-85-5** | Mirex | ClC12C(Cl)(Cl)C3(C4(C1(Cl)C1(C2(Cl)C3(C4(C1(Cl)Cl)Cl)Cl)Cl)Cl)Cl | 0.371 | 1.549 |
| **2437-79-8** | 2,2',4,4'-Tetrachlorobiphenyl | Clc1ccc(c(c1)Cl)c1ccc(cc1Cl)Cl | 0.833 | 0.370 |
| **2471-09-2** | OctacosamethylTridecasiloxane | C[Si](O[Si](O[Si](O[Si](O[Si](O[Si](O[Si](C)(C)C)(C)C)(C)C)(C)C)(C)C)(C)C)(O[Si](O[Si](O[Si](O[Si](O[Si](O[Si](C)(C)C)(C)C)(C)C)(C)C)(C)C)(C)C)C | -1.699 | 2.531 |
| **2471-10-5** | Triacontamethyltetradecasiloxane | C[Si](O[Si](O[Si](O[Si](O[Si](O[Si](O[Si](C)(C)C)(C)C)(C)C)(C)C)(C)C)(C)C)(O[Si](O[Si](O[Si](O[Si](O[Si](O[Si](O[Si](C)(C)C)(C)C)(C)C)(C)C)(C)C)(C)C)(C)C)C | -1.699 | 2.715 |
| **2652-13-3** | Eicosamethylnonasiloxane | C[Si](O[Si](O[Si](O[Si](O[Si](C)(C)C)(C)C)(C)C)(C)C)(O[Si](O[Si](O[Si](O[Si](C)(C)C)(C)C)(C)C)(C)C)C | -1.097 | 1.858 |
| **27575-78-6** | Tris(4-chlorophenyl)methane | Clc1ccc(cc1)C(c1ccc(cc1)Cl)c1ccc(cc1)Cl | 0.538 | 0.836 |
| **3010-80-8** | Tris(4-chlorophenyl)methanol | OC(c1ccc(cc1)Cl)(c1ccc(cc1)Cl)c1ccc(cc1)Cl | -0.260 | 0.997 |
| **31508-00-6** | 2,3',4,4',5-Pentachlorobiphenyl | Clc1cc(Cl)c(cc1c1ccc(c(c1)Cl)Cl)Cl | 0.776 | 0.531 |
| **32598-10-0** | 2,3',4,4'-Tetrachlorobiphenyl | Clc1ccc(c(c1)Cl)c1ccc(c(c1)Cl)Cl | 0.470 | 0.392 |
| **32598-11-1** | 2,3',4',5-Tetrachlorobiphenyl | Clc1ccc(c(c1)c1ccc(c(c1)Cl)Cl)Cl | 0.771 | 0.446 |
| **32598-14-4** | 2,3,3',4,4'-Pentachlorobiphenyl | Clc1ccc(cc1Cl)c1ccc(c(c1Cl)Cl)Cl | 0.740 | 0.406 |

| | | | | |
|---|---|---|---|---|
| **32690-93-0** | 2,4,4',5-Tetrachlorobiphenyl | Clc1ccc(cc1)c1cc(Cl)c(cc1Cl)Cl | 0.903 | 0.506 |
| **3322-93-8** | Tetrabromoethylcyclohexane β | BrCC(C1CCC(C(C1)Br)Br)Br | -0.318 | 0.239 |
| **33284-50-3** | 2,4-Dichlorobiphenyl | Clc1ccc(c(c1)Cl)c1ccccc1 | 0.703 | 0.170 |
| **33284-52-5** | 3,3',5,5'-Tetrachlorobiphenyl | Clc1cc(cc(c1)Cl)c1cc(Cl)cc(c1)Cl | 0.457 | 0.291 |
| **34123-59-6** | Isoproturon | O=C(N(C)C)Nc1ccc(cc1)C(C)C | -3.211 | -0.719 |
| **34883-39-1** | 2,5-Dichlorobiphenyl | Clc1ccc(c(c1)c1ccccc1)Cl | -0.638 | 0.268 |
| **35065-28-2** | 2,2',3,4,4',5'-Hexachlorobiphenyl | Clc1cc(Cl)c(cc1c1ccc(c(c1Cl)Cl)Cl)Cl | 0.803 | 0.577 |
| **35065-29-3** | 2,2',3,4,4',5,5'-Heptachlorobiphenyl | Clc1cc(Cl)c(cc1c1cc(Cl)c(c(c1Cl)Cl)Cl)Cl | 0.842 | 0.701 |
| **35065-30-6** | 2,2',3,3',4,4',5-Heptachlorobiphenyl | Clc1c(Cl)ccc(c1Cl)c1cc(Cl)c(c(c1Cl)Cl)Cl | 0.851 | 0.586 |
| **35693-99-3** | 2,2',5,5'-Tetrachlorobiphenyl | Clc1ccc(cc1c1cc(Cl)ccc1Cl)Cl | 0.481 | 0.549 |
| **35694-06-5** | 2,2',3,4,4',5-Hexachlorobiphenyl | Clc1ccc(c(c1)Cl)c1cc(Cl)c(c(c1Cl)Cl)Cl | 0.836 | 0.600 |
| **35694-08-7** | 2,2',3,3',4,4',5,5'-Octachlorobiphenyl | Clc1cc(c(c(c1Cl)Cl)Cl)c1cc(Cl)c(c(c1Cl)Cl)Cl | 0.958 | 0.669 |
| **35822-46-9** | 1,2,3,4,6,7,8-H7CDD | Clc1c(Cl)cc2c(c1Cl)Oc1c(O2)c(Cl)c(c(c1Cl)Cl)Cl | -0.469 | 0.644 |
| **36559-22-5** | 2,2',3,4'-Tetrachlorobiphenyl | Clc1ccc(c(c1)Cl)c1cccc(c1Cl)Cl | 0.572 | 0.334 |
| **36734-19-7** | Iprodione | CC(NC(=O)N1CC(=O)N(C1=O)c1cc(Cl)cc(c1)Cl)C | -2.673 | -0.835 |
| **37680-66-3** | 2,2',4-Trichlorobiphenyl | Clc1ccc(c(c1)Cl)c1ccccc1Cl | 0.204 | 0.250 |
| **37680-73-2** | 2,2',4,5,5'-Pentachlorobiphenyl | Clc1ccc(c(c1)c1cc(Cl)c(cc1Cl)Cl)Cl | 0.783 | 0.631 |
| **38379-99-6** | 2,2',3,5',6-Pentachlorobiphenyl | Clc1ccc(c(c1)c1c(Cl)ccc(c1Cl)Cl)Cl | 0.734 | 0.605 |
| **38380-02-8** | 2,2',3,4,5'-Pentachlorobiphenyl | Clc1ccc(c(c1)c1ccc(c(c1Cl)Cl)Cl)Cl | 0.760 | 0.506 |
| **38380-03-9** | 2,3,3',4',6-Pentachlorobiphenyl | Clc1cc(ccc1Cl)c1c(Cl)ccc(c1Cl)Cl | 0.919 | 0.381 |
| **38380-04-0** | 2,2',3,4',5',6-Hexachlorobiphenyl | Clc1cc(Cl)c(cc1c1c(Cl)ccc(c1Cl)Cl)Cl | 0.792 | 0.674 |
| **38380-05-1** | 2,2',3,3',4,6'-Hexachlorobiphenyl | Clc1ccc(c(c1Cl)Cl)c1c(Cl)ccc(c1Cl)Cl | 0.743 | 0.556 |
| **38380-07-3** | 2,2',3,3',4,4'-Hexachlorobiphenyl | Clc1c(ccc(c1Cl)Cl)c1ccc(c(c1Cl)Cl)Cl | 0.769 | 0.459 |
| **38380-08-4** | 2,3,3',4,4',5-Hexachlorobiphenyl | Clc1ccc(cc1Cl)c1cc(Cl)c(c(c1Cl)Cl)Cl | 0.869 | 0.528 |
| **38411-22-2** | 2,2',3,3',6,6'-Hexachlorobiphenyl | Clc1ccc(c(c1c1c(Cl)ccc(c1Cl)Cl)Cl)Cl | 0.678 | 0.771 |
| **38411-25-5** | 2,2',3,3',4,5,6'-Heptachlorobiphenyl | Clc1c(Cl)cc(c(c1Cl)Cl)c1c(Cl)ccc(c1Cl)Cl | 0.830 | 0.705 |
| **38444-76-7** | 2,3',6-Trichlorobiphenyl | Clc1cccc(c1)c1c(Cl)cccc1Cl | -0.046 | 0.062 |
| **38444-81-4** | 2,3',5-Trichlorobiphenyl | Clc1ccc(c(c1)c1cccc(c1)Cl)Cl | 0.653 | 0.333 |
| **38444-84-7** | 2,3,3'-Trichlorobiphenyl | Clc1cccc(c1)c1cccc(c1Cl)Cl | -0.222 | 0.173 |
| **38444-93-8** | 2,2',3,3'-Tetrachlorobiphenyl | Clc1c(Cl)cccc1c1cccc(c1Cl)Cl | 0.514 | 0.274 |
| **39227-61-7** | 1,2,3,4,7-P5CDD | Clc1ccc2c(c1)Oc1c(O2)c(Cl)c(c(c1Cl)Cl)Cl | -1.092 | 0.703 |
| **39635-31-9** | 2,3,3',4,4',5,5'-Heptachlorobiphenyl | Clc1cc(cc(c1Cl)Cl)c1cc(Cl)c(c(c1Cl)Cl)Cl | 0.631 | 0.514 |
| **40186-70-7** | 2,2',3,3',4,5',6-Heptachlorobiphenyl | Clc1cc(Cl)c(c(c1)c1c(Cl)cc(c(c1Cl)Cl)Cl)Cl | 0.778 | 0.705 |
| **40186-71-8** | 2,2',3,3',4,5',6,6'-Octachlorobiphenyl | Clc1cc(Cl)c(c(c1Cl)Cl)c1c(Cl)c(Cl)cc(c1Cl)Cl | 0.803 | 0.954 |
| **40186-72-9** | 2,2',3,3',4,4',5,5',6-Nonachlorobiphenyl | Clc1cc(c(c(c1Cl)Cl)Cl)c1c(Cl)c(Cl)c(c(c1Cl)Cl)Cl | 0.531 | 0.804 |
| **41464-41-9** | 2,2',5,6'-Tetrachlorobiphenyl | Clc1ccc(c(c1)c1c(Cl)cccc1Cl)Cl | -0.398 | 0.437 |
| **41464-43-1** | 2,3,3',4'-Tetrachlorobiphenyl | Clc1cc(ccc1Cl)c1cccc(c1Cl)Cl | 0.667 | 0.309 |
| **41464-46-4** | 2,3',4',6-Tetrachlorobiphenyl | Clc1cc(ccc1Cl)c1c(Cl)cccc1Cl | 0.322 | 0.220 |
| **41464-47-5** | 2,2',3,6'-Tetrachlorobiphenyl | Clc1c(Cl)cccc1c1c(Cl)cccc1Cl | -0.398 | 0.299 |
| **41464-51-1** | 2,2',3',4,5-Pentachlorobiphenyl | Clc1cc(Cl)c(cc1c1cccc(c1Cl)Cl)Cl | 0.842 | 0.506 |

| | | | | |
|---|---|---|---|---|
| **42740-50-1** | 2,2',3,3',4,4',5,6'-Octachlorobiphenyl | Clc1cc(Cl)c(c(c1c1cc(Cl)c(c(c1Cl)Cl)Cl)Cl)Cl | 0.833 | 0.741 |
| **483-65-8** | 1-methyl-7-(1methylethyl)-phenanthrene | CC(c1ccc2c(c1)ccc1c2cccc1C)C | -1.553 | -1.545 |
| **50-29-3** | 4,4'-DDT | ClC(C(c1ccc(cc1)Cl)c1ccc(cc1)Cl)(Cl)Cl | 0.752 | -0.009 |
| **50563-36-5** | Dimethachlor | COCCN(c1c(C)cccc1C)C(=O)CCl | -4.462 | -0.420 |
| **5103-74-2** | 1,2,4,5,6,7,8,8-Octachloro-2,3,3a,4,7,7a-Hexahydro-4,7-methano-1H-indene | ClC1CC2C(C1Cl)C1(C(C2(Cl)C(=C1Cl)Cl)(Cl)Cl)Cl | 0.326 | 0.645 |
| **51908-16-8** | 2,2',3,4',5,5'-Hexachlorobiphenyl | Clc1cc(Cl)c(c(c1)c1cc(Cl)c(cc1Cl)Cl)Cl | 0.916 | 0.649 |
| **52663-58-8** | 2,3,4',6-Tetrachlorobiphenyl | Clc1ccc(cc1)c1c(Cl)ccc(c1Cl)Cl | 0.806 | 0.310 |
| **52663-60-2** | 2,2',3,3',6-Pentachlorobiphenyl | Clc1c(Cl)cccc1c1c(Cl)ccc(c1Cl)Cl | 0.505 | 0.480 |
| **52663-61-3** | 2,2',3,5,5'-Pentachlorobiphenyl | Clc1ccc(c(c1)c1cc(Cl)cc(c1Cl)Cl)Cl | 0.744 | 0.579 |
| **52663-62-4** | 2,2',3,3',4-Pentachlorobiphenyl | Clc1ccc(c(c1Cl)Cl)c1cccc(c1Cl)Cl | 0.738 | 0.381 |
| **52663-65-7** | 2,2',3,3',4,6,6'-Heptachlorobiphenyl | Clc1cc(Cl)c(c(c1Cl)Cl)c1c(Cl)ccc(c1Cl)Cl | 0.767 | 0.823 |
| **52663-66-8** | 2,2',3,3',4,5'-Hexachlorobiphenyl | Clc1cc(Cl)c(c(c1)c1ccc(c(c1Cl)Cl)Cl)Cl | 0.431 | 0.530 |
| **52663-68-0** | 2,2',3,4',5,5',6-Heptachlorobiphenyl | Clc1cc(Cl)c(cc1c1c(Cl)c(Cl)cc(c1Cl)Cl)Cl | 0.756 | 0.798 |
| **52663-69-1** | 2,2',3,4,4',5',6-Heptachlorobiphenyl | Clc1cc(Cl)c(cc1c1c(Cl)cc(c(c1Cl)Cl)Cl)Cl | 0.790 | 0.727 |
| **52663-70-4** | 2,2',3,3',4,5',6'-Heptachlorobiphenyl | Clc1ccc(c(c1Cl)Cl)c1c(Cl)c(Cl)cc(c1Cl)Cl | 0.796 | 0.682 |
| **52663-71-5** | 2,2',3,3',4,4',6-Heptachlorobiphenyl | Clc1c(Cl)cc(c(c1Cl)c1ccc(c(c1Cl)Cl)Cl)Cl | 0.848 | 0.611 |
| **52663-72-6** | 2,3',4,4',5,5'-Hexachlorobiphenyl | Clc1cc(Cl)c(cc1c1cc(Cl)c(c(c1)Cl)Cl)Cl | 0.732 | 0.552 |
| **52663-74-8** | 2,2',3,3',4,5,5'-Heptachlorobiphenyl | Clc1cc(Cl)c(c(c1)c1cc(Cl)c(c(c1Cl)Cl)Cl)Cl | 0.854 | 0.633 |
| **52663-75-9** | 2,2',3,3',4,5,5',6'-Octachlorobiphenyl | Clc1c(Cl)cc(c(c1Cl)Cl)c1c(Cl)c(Cl)cc(c1Cl)Cl | 0.820 | 0.789 |
| **52663-77-1** | 2,2',3,3',4,5,5',6,6'-Nonachlorobiphenyl | Clc1c(Cl)c(Cl)c(c(c1Cl)Cl)c1c(Cl)c(Cl)cc(c1Cl)Cl | 0.728 | 1.048 |
| **52663-78-2** | 2,2',3,3',4,4',5,6-Octachlorobiphenyl | Clc1ccc(c(c1Cl)Cl)c1c(Cl)c(Cl)c(c(c1Cl)Cl)Cl | 0.641 | 0.717 |
| **52663-79-3** | 2,2',3,3',4,4',5,6,6'-Nonachlorobiphenyl | Clc1c(Cl)cc(c(c1Cl)c1c(Cl)c(Cl)c(c(c1Cl)Cl)Cl)Cl | 0.799 | 0.975 |
| **52712-04-6** | 2,2',3,4,5,5'-Hexachlorobiphenyl | Clc1ccc(c(c1)c1cc(Cl)c(c(c1Cl)Cl)Cl)Cl | 0.839 | 0.649 |
| **52712-05-7** | 2,2',3,4,5,5',6-Heptachlorobiphenyl | Clc1ccc(c(c1)c1c(Cl)c(Cl)c(c(c1Cl)Cl)Cl)Cl | 0.845 | 0.798 |
| **52744-13-5** | 2,2',3,3',5,6'-Hexachlorobiphenyl | Clc1cc(Cl)c(c(c1)c1c(Cl)ccc(c1Cl)Cl)Cl | 0.724 | 0.651 |
| **53-19-0** | o,p'-DDD | ClC(C(c1ccccc1Cl)c1ccc(cc1)Cl)Cl | 0.380 | 0.056 |
| **541-01-5** | Hexadecamethylheptasiloxane | C[Si](O[Si](O[Si](O[Si](C)(C)C)(C)C)(C)C)(O[Si](O[Si](O[Si](C)(C)C)(C)C)(C)C)C | -1.398 | 1.546 |
| **544-76-3** | n-Hexadecane | CCCCCCCCCCCCCCCC | -0.314 | -1.040 |
| **55215-18-4** | 2,2',3,3',4,5-Hexachlorobiphenyl | Clc1cccc(c1Cl)c1cc(Cl)c(c(c1Cl)Cl)Cl | 0.839 | 0.530 |
| **556-69-4** | Octadecamethyloctasiloxane | C[Si](O[Si](O[Si](O[Si](C)(C)C)(C)C)(C)C)(O[Si](O[Si](O[Si](O[Si](C)(C)C)(C)C)(C)C)(C)C)C | -0.796 | 1.698 |
| **556-70-7** | Docosamethyldecasiloxane | C[Si](O[Si](O[Si](O[Si](O[Si](C)(C)C)(C)C)(C)C)(C)C)(O[Si](O[Si](O[Si](O[Si](O[Si](C)(C)C)(C)C)(C)C)(C)C)(C)C)C | -1.222 | 2.023 |
| **56-55-3** | Benzo[a]anthracene | c1ccc2c(c1)cc1c(c2)ccc2c1cccc2 | -2.301 | -1.614 |
| **60145-20-2** | 2,2',3,3',5-Pentachlorobiphenyl | Clc1cc(Cl)c(c(c1)c1cccc(c1Cl)Cl)Cl | 0.761 | 0.454 |
| **60145-23-5** | 2,2',3,4,4',5,6'-Heptachlorobiphenyl | Clc1cc(Cl)c(c(c1)Cl)c1cc(Cl)c(c(c1Cl)Cl)Cl | 0.836 | 0.658 |
| **60233-25-2** | 2,2',3,4',6'-Pentachlorobiphenyl | Clc1cc(Cl)c(c(c1)Cl)c1cccc(c1Cl)Cl | 0.230 | 0.406 |
| **67129-08-2** | Metazachlor | ClCC(=O)N(c1c(C)cccc1C)Cn1cccn1 | -4.493 | -0.884 |

| | | | | |
|---|---|---|---|---|
| **68194-14-9** | 2,2',3,4,5',6-Hexachlorobiphenyl | Clc1ccc(c(c1)c1c(Cl)cc(c(c1Cl)Cl)Cl)Cl | 0.799 | 0.674 |
| **7012-37-5** | 2,4,4'-Trichlorobiphenyl | Clc1ccc(cc1)c1ccc(cc1Cl)Cl | 0.290 | 0.322 |
| **70362-45-7** | 2,2',3,6-Tetrachlorobiphenyl | Clc1ccccc1c1c(Cl)ccc(c1Cl)Cl | 0.568 | 0.414 |
| **70362-46-8** | 2,2',3,5-Tetrachlorobiphenyl | Clc1cc(Cl)c(c(c1)c1ccccc1Cl)Cl | 0.829 | 0.435 |
| **70424-68-9** | 2,3,3',4',5-Pentachlorobiphenyl | Clc1cc(Cl)c(c(c1)c1ccc(c(c1)Cl)Cl)Cl | 1.033 | 0.457 |
| **70424-70-3** | 2,3',4',5,5'-Pentachlorobiphenyl | Clc1ccc(c(c1)c1cc(Cl)c(c(c1)Cl)Cl)Cl | 0.380 | 0.480 |
| **71888-89-6** | Diisoheptyl phthalate | CC(CCCCOC(=O)c1ccccc1C(=O)OCCCCC(C)C)C | -2.509 | -1.121 |
| **72-54-8** | p,p'-DDD | ClC(C(c1ccc(cc1)Cl)c1ccc(cc1)Cl)Cl | 0.602 | 0.023 |
| **74472-33-6** | 2,3,3',6-Tetrachlorobiphenyl | Clc1cccc(c1)c1c(Cl)ccc(c1Cl)Cl | 0.720 | 0.274 |
| **74472-34-7** | 2,3,4',5-Tetrachlorobiphenyl | Clc1ccc(cc1)c1cc(Cl)cc(c1Cl)Cl | 0.857 | 0.446 |
| **74472-37-0** | 2,3,4,4',5-Pentachlorobiphenyl | Clc1ccc(cc1)c1cc(Cl)c(c(c1Cl)Cl)Cl | 0.829 | 0.531 |
| **74472-42-7** | 2,3,3',4,4',6-Hexachlorobiphenyl | Clc1ccc(cc1Cl)c1c(Cl)cc(c(c1Cl)Cl)Cl | 0.826 | 0.459 |
| **74472-44-9** | 2,3,3',4',5,6-Hexachlorobiphenyl | Clc1cc(ccc1Cl)c1c(Cl)c(Cl)cc(c1Cl)Cl | 0.929 | 0.507 |
| **74472-53-0** | 2,3,3',4,4',5,5',6-Octachlorobiphenyl | Clc1c(Cl)cc(cc1Cl)c1c(Cl)c(Cl)c(c(c1Cl)Cl)Cl | 0.806 | 0.552 |
| **779-02-2** | 9-Methylanthracene | Cc1c2ccccc2cc2c1cccc2 | -2.398 | -1.425 |
| **789-02-6** | 1-Chloro-2-(2,2,2-Trichloro-1-(4-chlorophenyl)ethyl)benzene (o,p'-DDT) | Clc1ccc(cc1)C(C(Cl)(Cl)Cl)c1ccccc1Cl | 0.839 | 0.114 |
| **79277-27-3** | Thifensulfuron-methyl | COc1nc(nc(n1)C)NC(=O)NS(=O)(=O)c1ccsc1C(=O)OC | -4.297 | -1.816 |
| **81777-89-1** | Clomazone | O=C1N(OCC1(C)C)Cc1ccccc1Cl | -3.699 | 0.543 |
| **83164-33-4** | Diflufenican | Fc1ccc(c(c1)F)NC(=O)c1cccnc1Oc1cccc(c1)C(F)(F)F | -2.991 | -0.847 |
| **84632-59-7** | 3,6-Bis-(4-tert-butyl-phenyl)-2,5-dihydro-pyrrolo[3,4-c]pyrrole-1,4-dione | CC(c1ccc(cc1)c1[nH]c(=O)c2c1c(=O)[nH]c2c1ccc(cc1)C(C)(C)C)(C)C | -1.413 | -1.228 |

**Table S3** – Dataset 3: Consistent dataset based on high quality data (Dataset 1) and verified low quality data from Dataset 2. Chemicals are reported with CAS numbers, names, splitting category (Training or Prediction set), experimental and predicted Log $BMF_L$, standardized residuals, HAT values and values of the molecular descriptors included in the model.

| CAS | Chemical Name | Split. Eq. 3 | Exp $BMF_L$ | Pred $BMF_L$ | Pred.Mod. Eq.Res. | Std.Pred. Mod.Eq. Res. | HAT i/i (h*=0.12) | PubchemFP503 | SubFPC295 | R_TpiPCTPC | MLFER_S | maxHother | GGI5 | VE3_Dt |
|---|---|---|---|---|---|---|---|---|---|---|---|---|---|---|
| **4390-04-9** | 2,2,4,4,6,8,8-heptamethyl nonane | Training | 0.004 | -0.513 | -0.518 | -1.247 | 0.093 | 0 | 0 | 1.000 | -0.149 | 0 | 0.778 | -4.765 |
| **1024-57-3** | 2,3,4,5,6,7,7-Heptachloro-1a,1b,5,5a,6,6a,-Hexahydro-(2a alpha, 1b beta, 2 alpha, 5 alpha, 5a beta, 6 beta, 6a alpha-2,5-methano-2H-indeno[1,2-b]oxirene | Training | 0.415 | -0.352 | -0.767 | -1.851 | 0.095 | 0 | 2 | 1.387 | 1.581 | 0 | 0.789 | -7.426 |
| **10394-57-7** | 9-n butylphenanthrene | Training | -1.854 | -1.512 | 0.342 | 0.795 | 0.024 | 0 | 0 | 15.938 | 1.237 | 0.43540529 | 0.389 | -3.214 |
| **104-72-3** | Decyl benzene | Prediction | -0.745 | -1.246 | -0.501 | -1.180 | 0.049 | 0 | 0 | 2.553 | 0.553 | 0.41190382 | 0.090 | -2.872 |
| **107534-96-3** | Tebuconazole | Training | -1.921 | -1.648 | 0.273 | 0.668 | 0.119 | 1 | 6 | 3.259 | 1.415 | 0.68678148 | 0.893 | -3.078 |
| **111-84-2** | n-nonane | Training | -0.893 | -0.734 | 0.159 | 0.371 | 0.034 | 0 | 0 | 1.000 | 0.127 | 0 | 0.056 | -30.905 |
| **112281-77-3** | Tetraconazole | Prediction | -2.046 | -2.215 | -0.169 | -0.425 | 0.169 | 1 | 7 | 3.957 | 1.406 | 0.75234702 | 0.847 | -2.903 |
| **112-40-3** | n-dodecane | Prediction | -0.878 | -0.850 | 0.028 | 0.065 | 0.044 | 0 | 0 | 1.000 | 0.127 | 0 | 0.056 | 0.000 |
| **112-41-4** | 1-dodecene | Training | -0.941 | -1.352 | -0.410 | -0.965 | 0.047 | 0 | 0 | 1.130 | 0.167 | 0.37125584 | 0.056 | 0.000 |
| **117-81-7** | Di-2-Ethylhexyl phthalate | Prediction | -1.125 | -2.541 | -1.416 | -3.463 | 0.119 | 0 | 6 | 3.007 | 1.313 | 0.55822961 | 0.917 | -99.355 |

| | | | | | | | | | | | | | | |
|---|---|---|---|---|---|---|---|---|---|---|---|---|---|---|
| **118-74-1** | Hexachlorobenzene | Training | 0.306 | -0.461 | -0.767 | -1.810 | 0.054 | 0 | 0 | 3.571 | 0.889 | 0 | 0.000 | -41.666 |
| **118-82-1** | Binox M | Prediction | 0.155 | 0.201 | 0.046 | 0.145 | 0.471 | 0 | 2 | 5.877 | 1.163 | 0.34168828 | 2.542 | -110.478 |
| **120068-37-3** | Fipronil | Training | -1.699 | -0.896 | 0.803 | 1.979 | 0.133 | 1 | 7 | 11.297 | 3.629 | 0.72210054 | 1.567 | -5.977 |
| **120-12-7** | Anthracene | Training | -1.875 | -1.871 | 0.004 | 0.009 | 0.036 | 0 | 0 | 18.797 | 1.261 | 0.45 | 0.111 | -3.781 |
| **123-48-8** | 2,2,4,4,6,6 pentamethyl-3-heptene | Training | -1.284 | -0.746 | 0.538 | 1.263 | 0.043 | 0 | 0 | 1.449 | 0.045 | 0.20298611 | 0.500 | -44.916 |
| **125116-23-6** | Metconazole | Training | -1.721 | -1.571 | 0.151 | 0.365 | 0.103 | 1 | 6 | 2.956 | 1.526 | 0.68167944 | 0.858 | -6.290 |
| **126690-66-2** | 2,4,6 Trimethyl-3-heptene | Prediction | -1.041 | -0.971 | 0.070 | 0.162 | 0.027 | 0 | 0 | 1.436 | 0.125 | 0.24076389 | 0.222 | -35.127 |
| **129-00-0** | Pyrene | Training | -2.301 | -1.862 | 0.439 | 1.046 | 0.071 | 0 | 0 | 24.028 | 1.503 | 0.45 | 0.111 | -54.942 |
| **13029-08-8** | 2,2'-Dichlorophenyl | Training | 0.663 | 0.208 | -0.455 | -1.064 | 0.035 | 1 | 0 | 11.992 | 1.285 | 0.43138203 | 0.194 | -48.012 |
| **13150-81-7** | 2,6-Dimethyldecane | Prediction | -1.319 | -0.762 | 0.557 | 1.306 | 0.042 | 0 | 0 | 1.000 | 0.049 | 0 | 0.222 | -6.058 |
| **13151-34-3** | 3-methyl decane | Prediction | -1.699 | -0.825 | 0.874 | 2.049 | 0.042 | 0 | 0 | 1.000 | 0.088 | 0 | 0.111 | -2.673 |
| **13475-82-6** | 2,2,4,6,6-pentamethylheptane | Training | -0.728 | -0.509 | 0.219 | 0.516 | 0.052 | 0 | 0 | 1.000 | -0.07 | 0 | 0.500 | -42.581 |
| **14233-37-5** | 9,10-Anthracenedione, 1,4-bis[(1-methylethyl)amino]- | Training | -0.500 | -1.994 | -1.493 | -3.633 | 0.110 | 0 | 6 | 6.900 | 2.239 | 0.54155286 | 1.389 | -82.012 |
| **15254-25-8** | 2,3,6,7-Tetramethylanthracene | Training | -1.757 | -1.401 | 0.356 | 0.836 | 0.046 | 0 | 0 | 18.211 | 1.165 | 0.41611111 | 0.444 | -63.229 |
| **15299-99-7** | Napropamide | Training | -2.852 | -3.083 | -0.231 | -0.554 | 0.089 | 0 | 6 | 7.742 | 1.935 | 0.52441397 | 0.486 | -1.468 |
| **15968-05-5** | 2,2',6,6'-Tetrachlorobiphenyl | Training | 0.623 | 0.441 | -0.183 | -0.427 | 0.036 | 1 | 0 | 11.576 | 1.475 | 0.40445169 | 0.250 | -55.555 |

| | | | | | | | | | | | | | |
|---|---|---|---|---|---|---|---|---|---|---|---|---|---|
| **1610-22-6** | 1,2,3,4,4a,7,8,9,10,11,12,12a-dodecahydrochrysene | Training | -0.770 | -0.569 | 0.200 | 0.472 | 0.049 | 0 | 0 | 3.137 | 0.853 | 0.30941185 | 0.361 | -62.901 |
| **16435-49-7** | 2-methyl-1-dodecene | Training | -1.200 | -1.275 | -0.075 | -0.176 | 0.041 | 0 | 0 | 1.124 | 0.143 | 0.34347806 | 0.111 | -3.938 |
| **16606-02-3** | 2,4',5-Trichlorobiphenyl | Prediction | 0.525 | 0.254 | -0.271 | -0.628 | 0.014 | 1 | 0 | 12.277 | 1.418 | 0.41421382 | 0.389 | -4.806 |
| **1678-98-4** | iso-butyl cyclohexane | Training | -0.652 | -0.763 | -0.111 | -0.261 | 0.040 | 0 | 0 | 1.000 | 0.199 | 0 | 0.118 | -1.567 |
| **16958-92-2** | Diisotridecyl adipate | Training | -1.959 | -2.172 | -0.213 | -0.573 | 0.273 | 0 | 6 | 1.114 | 0.989 | 0 | 0.222 | -129.757 |
| **1705-85-7** | 6-methylchyrsene | Training | -1.398 | -1.600 | -0.202 | -0.475 | 0.046 | 0 | 0 | 22.968 | 1.579 | 0.44609375 | 0.618 | -6.362 |
| **1706-50-9** | 1,1':3',1"Tercyclohexane | Training | -0.357 | -0.152 | 0.205 | 0.478 | 0.037 | 0 | 0 | 1.000 | 0.421 | 0 | 0.500 | -64.879 |
| **17088-22-1** | 1-ethylpyrene | Training | -1.699 | -1.750 | -0.051 | -0.120 | 0.045 | 0 | 0 | 22.804 | 1.479 | 0.44300733 | 0.458 | -5.362 |
| **17301-23-4** | 2,6-Dimethyl undecane | Training | -1.027 | -0.731 | 0.296 | 0.694 | 0.043 | 0 | 0 | 1.000 | 0.049 | 0 | 0.278 | -4.472 |
| **17312-44-6** | 2,3 Dimethyldecane | Prediction | -1.585 | -0.812 | 0.773 | 1.814 | 0.043 | 0 | 0 | 1.000 | 0.049 | 0 | 0.167 | -2.336 |
| **1732-13-4** | 1,2,3,6,7,8 Hexahydropyrene | Training | -1.252 | -0.986 | 0.266 | 0.625 | 0.043 | 0 | 0 | 8.114 | 1.123 | 0.36402778 | 0.111 | -56.051 |
| **1746-01-6** | 2,3,7,8-Tetrachlorodibenzo[b,e][1,4] Dioxin | Training | 0.103 | -0.391 | -0.495 | -1.182 | 0.078 | 1 | 4 | 7.554 | 2.419 | 0.52262086 | 0.444 | -63.229 |
| **1795-15-9** | n - Octyl Cyclohexane | Prediction | -1.222 | -0.756 | 0.466 | 1.091 | 0.040 | 0 | 0 | 1.000 | 0.238 | 0 | 0.090 | -2.449 |
| **18094-01-4** | 2 methyl 1-Tridecene | Prediction | -0.921 | -1.270 | -0.350 | -0.819 | 0.041 | 0 | 0 | 1.110 | 0.143 | 0.34199877 | 0.111 | -4.357 |
| **1836-75-5** | 2,4-Dichloro-1-(4-nitrophenoxy) benzene | Training | -0.821 | -0.855 | -0.034 | -0.080 | 0.040 | 1 | 3 | 7.060 | 1.539 | 0.64065558 | 0.417 | -4.690 |

| | | | | | | | | | | | | | | |
|---|---|---|---|---|---|---|---|---|---|---|---|---|---|---|
| **1839-63-0** | 1,3,5-Trimethyl cyclohexane | Training | -0.456 | -0.739 | -0.283 | -0.662 | 0.035 | 0 | 0 | 1.000 | 0.16 | 0 | 0.000 | -34.311 |
| **18516-37-5** | 2-methyl-1-undecene | Training | -1.456 | -1.280 | 0.176 | 0.413 | 0.041 | 0 | 0 | 1.141 | 0.143 | 0.3452 1417 | 0.111 | -3.532 |
| **1889-67-4** | Benzene, 1,1'-(1,1,2,2-tetramethyl-1,2-ethaneDiyl)bis- | Training | -0.777 | -0.654 | 0.123 | 0.293 | 0.069 | 0 | 0 | 4.834 | 0.821 | 0.4089 6259 | 0.625 | -63.275 |
| **191-24-2** | Benzo(ghi)perylene | Prediction | -1.495 | -1.362 | 0.133 | 0.323 | 0.110 | 0 | 0 | 29.450 | 2.087 | 0.45 | 0.736 | -79.929 |
| **193-39-5** | Indeno 1,2,3-cd pyrene | Prediction | -1.538 | -1.560 | -0.022 | -0.053 | 0.083 | 0 | 0 | 29.487 | 2.087 | 0.45 | 0.820 | -13.214 |
| **194-69-4** | Benzo(c)chrysene | Training | -1.301 | -1.655 | -0.354 | -0.839 | 0.059 | 0 | 0 | 26.325 | 1.945 | 0.45 | 0.542 | -6.220 |
| **19780-74-6** | 5-ethyl-1-nonene | Prediction | -0.461 | -1.149 | -0.688 | -1.618 | 0.048 | 0 | 0 | 1.152 | 0.128 | 0.3655 1158 | 0.167 | -38.756 |
| **20279-21-4** | 1,2,3,10b-Tetrahydrofluoranthene | Training | -1.638 | -1.187 | 0.451 | 1.055 | 0.038 | 0 | 0 | 9.009 | 1.208 | 0.4228 5147 | 0.219 | -7.015 |
| **204256-07-5** | 2,3 Dimethy-5(4methylpentyl) naphthalene | Training | -1.367 | -1.194 | 0.172 | 0.402 | 0.030 | 0 | 0 | 8.907 | 0.808 | 0.4009 2998 | 0.514 | -3.379 |
| **2050-68-2** | 4,4'-Dichlorobiphenyl | Training | 0.519 | 0.283 | -0.235 | -0.549 | 0.033 | 1 | 0 | 12.813 | 1.285 | 0.4272 2584 | 0.361 | -50.864 |
| **2051-24-3** | Decachlorobiphenyl | Training | 0.319 | 0.392 | 0.073 | 0.177 | 0.107 | 0 | 0 | 11.633 | 1.701 | 0 | 1.139 | -79.929 |
| **2051-30-1** | 2,6-Dimethyl octane | Prediction | -1.854 | -0.770 | 1.084 | 2.542 | 0.043 | 0 | 0 | 1.000 | 0.049 | 0 | 0.222 | -3.756 |
| **205646-11-3** | 1,2,9,10-Tetrachlorodecane | Training | -0.658 | -0.285 | 0.372 | 0.871 | 0.038 | 0 | 0 | 1.000 | 0.677 | 0 | 0.167 | -49.491 |
| **206-44-0** | Fluoranthene | Training | -1.553 | -1.894 | -0.341 | -0.801 | 0.047 | 0 | 0 | 22.751 | 1.503 | 0.45 | 0.219 | -7.015 |
| **207-08-9** | Benzo(k)fluoranthene | Prediction | -1.979 | -1.696 | 0.282 | 0.669 | 0.062 | 0 | 0 | 26.583 | 1.845 | 0.45 | 0.594 | -5.116 |
| **2136-99-4** | 2,2',3,3',5,5',6,6'- | Prediction | 0.668 | 1.217 | 0.550 | 1.296 | 0.052 | 1 | 0 | 11.451 | 1.759 | 0.3348 2506 | 0.917 | -76.246 |

| | | | | | | | | | | | | | | |
|---|---|---|---|---|---|---|---|---|---|---|---|---|---|---|
| | Octachlorobiphenyl | | | | | | | | | | | | | |
| **214-17-5** | Benzo[b]chrysene | Training | -1.745 | -1.679 | 0.066 | 0.156 | 0.060 | 0 | 0 | 26.594 | 1.945 | 0.45 | 0.528 | -6.755 |
| **217-59-4** | Triphenylene | Training | -1.658 | -1.517 | 0.141 | 0.333 | 0.060 | 0 | 0 | 22.798 | 1.603 | 0.45 | 0.375 | -66.126 |
| **218-01-9** | Chrysene | Training | -1.538 | -1.577 | -0.039 | -0.093 | 0.063 | 0 | 0 | 23.536 | 1.603 | 0.45 | 0.361 | -64.477 |
| **2189-60-8** | n-octyl benzene | Prediction | -1.648 | -1.265 | 0.383 | 0.900 | 0.048 | 0 | 0 | 2.788 | 0.553 | 0.41465862 | 0.090 | -2.449 |
| **221155-23-3** | 1,2,13,14-Tetrachlorotetradecane | Training | 0.100 | -0.228 | -0.328 | -0.770 | 0.044 | 0 | 0 | 1.000 | 0.677 | 0 | 0.167 | -64.879 |
| **22907-72-8** | 1,4 Diisopropyl cyclohexane | Training | -0.479 | -0.495 | -0.017 | -0.039 | 0.034 | 0 | 0 | 1.000 | 0.121 | 0 | 0.361 | -42.153 |
| **23342-25-8** | 2,2,5,7-Tetramethyltetraline | Training | -1.301 | -0.916 | 0.385 | 0.903 | 0.041 | 0 | 0 | 3.528 | 0.552 | 0.34167092 | 0.535 | -3.084 |
| **2381-21-7** | 1-Methylpyrene | Training | -2.301 | -1.853 | 0.448 | 1.055 | 0.049 | 0 | 0 | 23.535 | 1.479 | 0.44609375 | 0.368 | -6.606 |
| **243-17-4** | Benzo(b)fluorene | Training | -1.602 | -1.404 | 0.198 | 0.460 | 0.027 | 0 | 0 | 16.747 | 1.463 | 0.44489796 | 0.445 | -5.006 |
| **2435-85-0** | Hexadecahydropyrene | Training | 0.009 | -0.404 | -0.412 | -0.964 | 0.035 | 0 | 0 | 1.000 | 0.493 | 0 | 0.111 | -54.942 |
| **2437-56-1** | 1-Tridecene | Training | -0.963 | -1.177 | -0.214 | -0.505 | 0.050 | 0 | 0 | 1.114 | 0.167 | 0.36977655 | 0.056 | -45.803 |
| **2437-79-8** | 2,2',4,4'-Tetrachlorobiphenyl | Training | 0.833 | 0.520 | -0.313 | -0.729 | 0.029 | 1 | 0 | 12.168 | 1.475 | 0.39906998 | 0.417 | -55.095 |
| **2541-69-7** | 7-Methylbenz(a)anthracene | Training | -2.097 | -1.626 | 0.471 | 1.107 | 0.045 | 0 | 0 | 23.021 | 1.579 | 0.44609375 | 0.583 | -6.404 |
| **25569-80-6** | 2,3'-Dichlorobiphenyl | Training | 0.267 | 0.128 | -0.140 | -0.323 | 0.018 | 1 | 0 | 12.170 | 1.285 | 0.4334064 | 0.333 | -6.032 |
| **26186-00-5** | 1-Heptadecyne | Training | -0.086 | -0.577 | -0.491 | -1.149 | 0.037 | 0 | 0 | 1.152 | 0.236 | 0 | 0.056 | -59.027 |
| **2732-58-3** | 6-ethylchyrsene | Training | -1.495 | -1.496 | -0.001 | -0.003 | 0.046 | 0 | 0 | 22.238 | 1.579 | 0.44300733 | 0.708 | -5.357 |
| **27575-78-6** | Tris(4-chlorophenyl) methane | Training | 0.538 | 0.989 | 0.451 | 1.075 | 0.072 | 1 | 0 | 8.126 | 1.765 | 0.39707124 | 0.375 | -78.404 |

| | | | | | | | | | | | | | | |
|---|---|---|---|---|---|---|---|---|---|---|---|---|---|---|
| **2974-92-7** | 3,4-Dichlorobiphenyl | Training | 0.477 | 0.080 | -0.397 | -0.920 | 0.019 | 1 | 0 | 12.466 | 1.242 | 0.4423167 | 0.361 | -3.075 |
| **2980-71-4** | 2-methyl-1-nonene | Training | -1.367 | -1.290 | 0.076 | 0.179 | 0.042 | 0 | 0 | 1.164 | 0.143 | 0.34978029 | 0.111 | -2.769 |
| **3010-80-8** | Tris(4-chlorophenyl) methanol | Training | -0.260 | 0.639 | 0.898 | 2.101 | 0.037 | 1 | 1 | 7.844 | 2.047 | 0.44334491 | 0.729 | 0.000 |
| **3074-71-3** | 2,3-Dimethylheptane | Prediction | -0.792 | -0.850 | -0.058 | -0.137 | 0.044 | 0 | 0 | 1.000 | 0.049 | 0 | 0.111 | -1.816 |
| **31508-00-6** | 2,3',4,4',5-Pentachlorobiphenyl | Training | 0.776 | 0.500 | -0.276 | -0.637 | 0.011 | 1 | 0 | 12.328 | 1.56 | 0.39762022 | 0.611 | -5.396 |
| **319-84-6** | (1 alpha,2 alpha,3 beta,4 alpha,5 beta,6 beta)1,2,3,4,5,6-Hexachlorocyclohexane | Training | -1.516 | -0.252 | 1.264 | 3.011 | 0.072 | 0 | 0 | 1.000 | 0.985 | 0 | 0.000 | -41.666 |
| **32598-10-0** | 2,3',4,4'-Tetrachlorobiphenyl | Training | 0.470 | 0.406 | -0.064 | -0.147 | 0.012 | 1 | 0 | 12.410 | 1.47 | 0.40525054 | 0.556 | -7.469 |
| **32598-11-1** | 2,3',4',5-Tetrachlorobiphenyl | Prediction | 0.771 | 0.408 | -0.363 | -0.839 | 0.011 | 1 | 0 | 12.196 | 1.508 | 0.40322617 | 0.500 | -7.727 |
| **32598-14-4** | 2,3,3',4,4'-Pentachlorobiphenyl | Training | 0.740 | 0.477 | -0.263 | -0.608 | 0.012 | 1 | 0 | 12.334 | 1.517 | 0.39762022 | 0.611 | -5.638 |
| **32690-93-0** | 2,4,4',5-Tetrachlorobiphenyl | Training | 0.903 | 0.356 | -0.547 | -1.264 | 0.012 | 1 | 0 | 12.341 | 1.47 | 0.40860787 | 0.500 | -3.883 |
| **32774-16-6** | 3,3',4,4',5,5'-Hexachlorobiphenyl | Training | 0.496 | 0.731 | 0.235 | 0.549 | 0.032 | 1 | 0 | 12.455 | 1.569 | 0.3704563 | 0.583 | -62.383 |
| **33025-41-1** | 2,3,4,4'-Tetrachlorobiphenyl | Training | 0.688 | 0.332 | -0.357 | -0.824 | 0.013 | 1 | 0 | 12.348 | 1.427 | 0.40860787 | 0.500 | -3.993 |
| **3321-50-4** | Cyclohexane, 1,1'-(1,2- | Training | -1.301 | -0.463 | 0.838 | 1.952 | 0.029 | 0 | 0 | 1.000 | 0.349 | 0 | 0.181 | -48.355 |

| | | | | | | | | | | | | | |
|---|---|---|---|---|---|---|---|---|---|---|---|---|---|
| | EthaneDiyl)Bis- | | | | | | | | | | | | |
| **3322-93-8** | Tetrabromoethylcyclohexane β | Training | -0.318 | -0.146 | 0.172 | 0.410 | 0.071 | 0 | 0 | 1.000 | 1.065 | 0 | 0.319 | -2.222 |
| **33284-50-3** | 2,4-Dichlorobiphenyl | Prediction | 0.703 | 0.041 | -0.663 | -1.537 | 0.021 | 1 | 0 | 12.304 | 1.247 | 0.4410028 | 0.278 | -3.177 |
| **33284-52-5** | 3,3',5,5'-Tetrachlorobiphenyl | Prediction | 0.457 | 0.496 | 0.039 | 0.090 | 0.031 | 1 | 0 | 12.125 | 1.475 | 0.39323045 | 0.361 | -55.555 |
| **33460-02-5** | 3 Phenyl bicyclohexyl | Training | -1.046 | -1.275 | -0.229 | -0.537 | 0.041 | 0 | 0 | 1.124 | 0.143 | 0.34347806 | 0.111 | -3.938 |
| **34883-39-1** | 2,5-Dichlorobiphenyl | Prediction | -0.638 | 0.077 | 0.715 | 1.657 | 0.020 | 1 | 0 | 12.078 | 1.285 | 0.440102 | 0.278 | -3.122 |
| **34883-43-7** | 2,4'-Dichlorobiphenyl | Prediction | 0.574 | 0.077 | -0.497 | -1.152 | 0.020 | 1 | 0 | 12.335 | 1.285 | 0.43472029 | 0.278 | -5.408 |
| **35065-27-1** | 2,2',4,4',5,5'-Hexachlorobiphenyl | Training | 0.614 | 0.899 | 0.285 | 0.665 | 0.034 | 1 | 0 | 12.085 | 1.655 | 0.3609184 | 0.694 | -65.396 |
| **35065-28-2** | 2,2',3,4,4',5'-Hexachlorobiphenyl | Training | 0.803 | 0.638 | -0.165 | -0.382 | 0.012 | 1 | 0 | 12.091 | 1.612 | 0.38045201 | 0.694 | -9.494 |
| **35065-29-3** | 2,2',3,4,4',5,5'-Heptachlorobiphenyl | Training | 0.842 | 0.767 | -0.075 | -0.173 | 0.016 | 1 | 0 | 12.031 | 1.664 | 0.35328809 | 0.806 | -5.902 |
| **35065-30-6** | 2,2',3,3',4,4',5-Heptachlorobiphenyl | Training | 0.851 | 0.715 | -0.136 | -0.315 | 0.016 | 1 | 0 | 12.036 | 1.621 | 0.3728217 | 0.806 | -5.703 |
| **35693-99-3** | 2,2',5,5'-Tetrachlorobiphenyl | Training | 0.481 | 0.640 | 0.158 | 0.369 | 0.030 | 1 | 0 | 11.865 | 1.551 | 0.39241253 | 0.472 | -58.779 |
| **35694-06-5** | 2,2',3,4,4',5-Hexachlorobiphenyl | Training | 0.836 | 0.629 | -0.206 | -0.477 | 0.014 | 1 | 0 | 12.037 | 1.574 | 0.38535682 | 0.750 | -4.217 |
| **35694-08-7** | 2,2',3,3',4,4',5,5'-Octachlorobiphenyl | Training | 0.958 | 1.076 | 0.118 | 0.277 | 0.045 | 1 | 0 | 11.997 | 1.673 | 0.34230043 | 0.861 | -72.663 |

| | | | | | | | | | | | | | |
|---|---|---|---|---|---|---|---|---|---|---|---|---|---|
| **35822-46-9** | 1,2,3,4,6,7,8-H7CDD | Training | -0.469 | -0.352 | 0.117 | 0.274 | 0.041 | 1 | 4 | 7.351 | 2.332 | 0.49839695 | 0.799 | -12.460 |
| **36559-22-5** | 2,2',3,4'-Tetrachlorobiphenyl | Training | 0.572 | 0.369 | -0.203 | -0.469 | 0.012 | 1 | 0 | 12.064 | 1.47 | 0.40992177 | 0.472 | -8.219 |
| **37680-65-2** | 2,2',5-Trichlorobiphenyl | Training | 0.580 | 0.239 | -0.341 | -0.788 | 0.015 | 1 | 0 | 11.910 | 1.418 | 0.42577608 | 0.361 | -4.155 |
| **37680-66-3** | 2,2',4-Trichlorobiphenyl | Training | 0.204 | 0.171 | -0.033 | -0.077 | 0.017 | 1 | 0 | 12.058 | 1.38 | 0.42708998 | 0.306 | -4.197 |
| **37680-73-2** | 2,2',4,5,5'-Pentachlorobiphenyl | Training | 0.783 | 0.539 | -0.244 | -0.563 | 0.012 | 1 | 0 | 11.961 | 1.603 | 0.38902128 | 0.583 | -5.018 |
| **38379-99-6** | 2,2',3,5',6-Pentachlorobiphenyl | Training | 0.734 | 0.542 | -0.192 | -0.443 | 0.012 | 1 | 0 | 11.670 | 1.603 | 0.38680658 | 0.556 | -5.258 |
| **38380-01-7** | 2,2',4,4',5-Pentachlorobiphenyl | Training | 0.868 | 0.505 | -0.363 | -0.838 | 0.011 | 1 | 0 | 12.115 | 1.565 | 0.39143966 | 0.583 | -5.030 |
| **38380-02-8** | 2,2',3,4,5'-Pentachlorobiphenyl | Prediction | 0.760 | 0.515 | -0.244 | -0.564 | 0.011 | 1 | 0 | 11.968 | 1.56 | 0.38902128 | 0.583 | -5.198 |
| **38380-03-9** | 2,3,3',4',6-Pentachlorobiphenyl | Prediction | 0.919 | 0.514 | -0.405 | -0.934 | 0.011 | 1 | 0 | 11.940 | 1.56 | 0.39223851 | 0.583 | -5.687 |
| **38380-04-0** | 2,2',3,4',5',6-Hexachlorobiphenyl | Training | 0.792 | 0.669 | -0.123 | -0.285 | 0.012 | 1 | 0 | 11.757 | 1.655 | 0.37379456 | 0.667 | -8.553 |
| **38380-05-1** | 2,2',3,3',4,6'-Hexachlorobiphenyl | Prediction | 0.743 | 0.649 | -0.094 | -0.217 | 0.012 | 1 | 0 | 11.763 | 1.612 | 0.3750703 | 0.667 | -10.083 |
| **38380-07-3** | 2,2',3,3',4,4'-Hexachlorobiphenyl | Training | 0.769 | 0.812 | 0.043 | 0.099 | 0.032 | 1 | 0 | 12.096 | 1.569 | 0.38045201 | 0.694 | -62.440 |
| **38380-08-4** | 2,3,3',4,4',5-Hexachlorobiphenyl | Training | 0.869 | 0.555 | -0.314 | -0.726 | 0.012 | 1 | 0 | 12.228 | 1.569 | 0.3899899 | 0.667 | -4.434 |
| **38411-22-2** | 2,2',3,3',6,6'-Hexachlorobiphenyl | Training | 0.678 | 0.882 | 0.204 | 0.477 | 0.034 | 1 | 0 | 11.520 | 1.655 | 0.3704563 | 0.639 | -65.119 |

| | | | | | | | | | | | | | | |
|---|---|---|---|---|---|---|---|---|---|---|---|---|---|---|
| **38411-25-5** | 2,2',3,3',4,5,6'-Heptachlorobiphenyl | Training | 0.830 | 0.784 | -0.047 | -0.108 | 0.017 | 1 | 0 | 11.693 | 1.664 | 0.36818861 | 0.833 | -5.512 |
| **38444-73-4** | 2,2',6-Trichlorobiphenyl | Training | 0.568 | 0.141 | -0.428 | -0.992 | 0.021 | 1 | 0 | 11.754 | 1.38 | 0.42375172 | 0.222 | -4.450 |
| **38444-76-7** | 2,3',6-Trichlorobiphenyl | Prediction | -0.046 | 0.268 | 0.313 | 0.724 | 0.014 | 1 | 0 | 11.928 | 1.38 | 0.41757116 | 0.417 | -4.920 |
| **38444-77-8** | 2,4',6-Trichlorobiphenyl | Training | 0.580 | 0.198 | -0.382 | -0.884 | 0.017 | 1 | 0 | 12.022 | 1.38 | 0.41227233 | 0.306 | -5.303 |
| **38444-78-9** | 2,2',3-Trichlorobiphenyl | Prediction | 0.477 | 0.215 | -0.262 | -0.605 | 0.015 | 1 | 0 | 11.918 | 1.375 | 0.42577608 | 0.361 | -4.326 |
| **38444-81-4** | 2,3',5-Trichlorobiphenyl | Training | 0.653 | 0.257 | -0.396 | -0.916 | 0.014 | 1 | 0 | 12.065 | 1.418 | 0.42039438 | 0.389 | -4.529 |
| **38444-84-7** | 2,3,3'-Trichlorobiphenyl | Training | -0.222 | 0.233 | 0.455 | 1.052 | 0.014 | 1 | 0 | 12.073 | 1.375 | 0.42039438 | 0.389 | -4.765 |
| **38444-85-8** | 2,3,4'-Trichlorobiphenyl | Training | 0.352 | 0.226 | -0.127 | -0.293 | 0.015 | 1 | 0 | 12.285 | 1.375 | 0.41755208 | 0.389 | -5.108 |
| **38444-86-9** | 2,3',4'-Trichlorobiphenyl | Training | 0.519 | 0.246 | -0.273 | -0.630 | 0.014 | 1 | 0 | 12.218 | 1.375 | 0.42911435 | 0.444 | -4.005 |
| **38444-93-8** | 2,2',3,3'-Tetrachlorobiphenyl | Training | 0.514 | 0.555 | 0.041 | 0.095 | 0.028 | 1 | 0 | 11.880 | 1.465 | 0.40860787 | 0.472 | -55.177 |
| **3891-98-3** | 2,6,10-Trimethyl dodecane | Training | -0.002 | -0.672 | -0.670 | -1.576 | 0.047 | 0 | 0 | 1.000 | 0.01 | 0 | 0.389 | -6.604 |
| **39227-61-7** | 1,2,3,4,7-P5CDD | Training | -1.092 | -0.545 | 0.547 | 1.285 | 0.046 | 1 | 4 | 7.391 | 2.309 | 0.53990586 | 0.653 | -6.003 |
| **39635-31-9** | 2,3,3',4,4',5,5'-Heptachlorobiphenyl | Prediction | 0.631 | 0.671 | 0.040 | 0.092 | 0.014 | 1 | 0 | 12.203 | 1.621 | 0.35946864 | 0.722 | -6.158 |
| **40186-70-7** | 2,2',3,3',4,5',6-Heptachlorobiphenyl | Training | 0.778 | 0.801 | 0.022 | 0.052 | 0.017 | 1 | 0 | 11.765 | 1.669 | 0.3561113 | 0.833 | -5.977 |

| | | | | | | | | | | | | | | |
|---|---|---|---|---|---|---|---|---|---|---|---|---|---|---|
| **40186-71-8** | 2,2',3,3',4,5',6,6'-Octachlorobiphenyl | Training | 0.803 | 0.948 | 0.145 | 0.338 | 0.021 | 1 | 0 | 11.587 | 1.721 | 0.33572586 | 0.917 | -12.645 |
| **40186-72-9** | 2,2',3,3',4,4',5,5',6-Nonachlorobiphenyl | Training | 0.532 | 0.955 | 0.423 | 0.985 | 0.027 | 1 | 0 | 11.797 | 1.687 | 0.33131278 | 1.000 | -6.678 |
| **40458-98-8** | 2,7-Diisopropyl naphthalene | Prediction | -1.438 | -1.025 | 0.413 | 0.968 | 0.041 | 0 | 0 | 9.992 | 0.793 | 0.39722647 | 0.583 | -54.871 |
| **41411-64-7** | 2,3,3',4,4',5,6-Heptachlorobiphenyl | Training | 0.894 | 0.648 | -0.246 | -0.569 | 0.014 | 1 | 0 | 11.947 | 1.583 | 0.37900225 | 0.750 | -3.931 |
| **41464-39-5** | 2,2',3,5'-Tetrachlorobiphenyl | Prediction | 0.789 | 0.403 | -0.386 | -0.891 | 0.012 | 1 | 0 | 11.873 | 1.508 | 0.40860787 | 0.472 | -8.059 |
| **41464-41-9** | 2,2',5,6'-Tetrachlorobiphenyl | Training | -0.398 | 0.397 | 0.795 | 1.835 | 0.012 | 1 | 0 | 11.729 | 1.513 | 0.40576558 | 0.444 | -7.135 |
| **41464-43-1** | 2,3,3',4'-Tetrachlorobiphenyl | Training | 0.668 | 0.367 | -0.300 | -0.693 | 0.012 | 1 | 0 | 12.203 | 1.465 | 0.41194614 | 0.500 | -6.728 |
| **41464-46-4** | 2,3',4',6-Tetrachlorobiphenyl | Prediction | 0.322 | 0.406 | 0.084 | 0.194 | 0.011 | 1 | 0 | 11.972 | 1.47 | 0.40798028 | 0.528 | -6.252 |
| **41464-47-5** | 2,2',3,6'-Tetrachlorobiphenyl | Training | -0.398 | 0.376 | 0.774 | 1.787 | 0.012 | 1 | 0 | 11.736 | 1.47 | 0.40658351 | 0.444 | -8.388 |
| **41464-49-7** | 2,3,3',5'-Tetrachlorobiphenyl | Training | 0.238 | 0.384 | 0.146 | 0.338 | 0.012 | 1 | 0 | 12.010 | 1.47 | 0.41063224 | 0.500 | -6.875 |
| **41464-51-1** | 2,2',3',4,5-Pentachlorobiphenyl | Training | 0.842 | 0.493 | -0.349 | -0.807 | 0.011 | 1 | 0 | 11.968 | 1.56 | 0.40431582 | 0.583 | -4.824 |
| **4175-54-6** | 1,2,3,4-Tetrahydro-1,4-Dimethyl naphthalene (1,4-Dimethyl Tetralin) | Training | -0.801 | -1.079 | -0.277 | -0.651 | 0.042 | 0 | 0 | 3.479 | 0.601 | 0.39048611 | 0.125 | -41.263 |

| | | | | | | | | | | | | | |
|---|---|---|---|---|---|---|---|---|---|---|---|---|---|
| **42740-50-1** | 2,2',3,3',4,4',5,6'-Octachlorobiphenyl | Prediction | 0.833 | 0.915 | 0.083 | 0.192 | 0.023 | 1 | 0 | 11.840 | 1.678 | 0.3389 4309 | 0.944 | -10.628 |
| **4292-75-5** | n-hexyl cyclohexane | Prediction | -1.377 | -0.758 | 0.619 | 1.451 | 0.040 | 0 | 0 | 1.000 | 0.238 | 0 | 0.090 | -2.103 |
| **43121-43-3** | Triadimefon | Prediction | -1.921 | -2.403 | -0.482 | -1.218 | 0.174 | 1 | 8 | 4.411 | 1.789 | 0.7449 3308 | 0.871 | -2.373 |
| **4316-65-8** | 3,5,5' Trimethyl hexene | Training | -1.201 | -1.433 | -0.232 | -0.545 | 0.046 | 0 | 0 | 1.178 | 0.049 | 0.3601 3889 | 0.000 | -2.286 |
| **438-22-2** | 5α(H)-androstane | Training | -0.172 | -0.271 | -0.099 | -0.232 | 0.047 | 0 | 0 | 1.000 | 0.413 | 0 | 0.667 | -5.322 |
| **475-03-6** | 1,1,6 Trimethyl Tetralin | Training | -0.484 | -1.064 | -0.580 | -1.356 | 0.035 | 0 | 0 | 3.480 | 0.576 | 0.3596 5278 | 0.319 | -3.409 |
| **483-65-8** | 1-methyl-7-(1methylethyl)-phenanthrene | Training | -1.553 | -1.488 | 0.065 | 0.151 | 0.031 | 0 | 0 | 17.081 | 1.174 | 0.4270 8546 | 0.563 | -3.852 |
| **488-23-3** | 1,2,3,4 Tetramethyl benzene | Prediction | -1.292 | -1.250 | 0.042 | 0.099 | 0.038 | 0 | 0 | 3.537 | 0.481 | 0.3809 7222 | 0.000 | -32.642 |
| **4920-95-0** | 3,3',4,4' Tetramethyl 1,1'-biphenyl | Training | -2.046 | -1.153 | 0.893 | 2.090 | 0.039 | 0 | 0 | 12.631 | 0.923 | 0.4123 2852 | 0.528 | -59.239 |
| **496-10-6** | Bicyclo[4.3.0] nonane | Prediction | -1.102 | -0.646 | 0.457 | 1.068 | 0.035 | 0 | 0 | 1.000 | 0.349 | 0 | 0.000 | -31.250 |
| **50-29-3** | 4,4'-DDT | Training | 0.752 | 1.045 | 0.293 | 0.689 | 0.047 | 1 | 0 | 5.938 | 1.448 | 0.3675 7154 | 0.528 | -67.420 |
| **50-32-8** | Benzo[a]pyrene | Training | -1.745 | -1.876 | -0.131 | -0.312 | 0.067 | 0 | 0 | 27.149 | 1.845 | 0.45 | 0.375 | -4.691 |
| **50876-32-9** | cis 1,1,3,5 Tetramethyl cyclohexane | Training | -0.265 | -0.881 | -0.616 | -1.446 | 0.045 | 0 | 0 | 1.000 | 0.12 | 0 | 0.000 | -2.468 |
| **5103-74-2** | 1,2,4,5,6,7,8,8-Octachloro-2,3,3a,4,7,7a-Hexahydro-4,7-methano-1H-indene | Training | 0.326 | 0.360 | 0.034 | 0.082 | 0.105 | 0 | 0 | 1.401 | 1.442 | 0 | 0.768 | -9.294 |

| | | | | | | | | | | | | | | |
|---|---|---|---|---|---|---|---|---|---|---|---|---|---|---|
| **51655-65-3** | 2-butyl-1-decene | Prediction | -0.975 | -1.237 | -0.262 | -0.612 | 0.037 | 0 | 0 | 1.127 | 0.143 | 0.31471085 | 0.111 | -3.544 |
| **51908-16-8** | 2,2',3,4',5,5'-Hexachlorobiphenyl | Training | 0.917 | 0.697 | -0.219 | -0.507 | 0.013 | 1 | 0 | 11.950 | 1.655 | 0.36709896 | 0.694 | -11.751 |
| **52663-58-8** | 2,3,4',6-Tetrachlorobiphenyl | Prediction | 0.806 | 0.332 | -0.474 | -1.096 | 0.013 | 1 | 0 | 11.962 | 1.47 | 0.40322617 | 0.417 | -3.967 |
| **52663-59-9** | 2,2',3,4-Tetrachlorobiphenyl | Training | 0.218 | 0.319 | 0.101 | 0.234 | 0.012 | 1 | 0 | 11.940 | 1.427 | 0.42148403 | 0.472 | -3.564 |
| **52663-60-2** | 2,2',3,3',6-Pentachlorobiphenyl | Prediction | 0.505 | 0.498 | -0.008 | -0.018 | 0.011 | 1 | 0 | 11.676 | 1.56 | 0.40097756 | 0.556 | -5.044 |
| **52663-61-3** | 2,2',3,5,5'-Pentachlorobiphenyl | Training | 0.744 | 0.551 | -0.194 | -0.448 | 0.012 | 1 | 0 | 11.793 | 1.603 | 0.38812048 | 0.583 | -5.038 |
| **52663-62-4** | 2,2',3,3',4-Pentachlorobiphenyl | Training | 0.738 | 0.469 | -0.269 | -0.621 | 0.011 | 1 | 0 | 11.974 | 1.517 | 0.40431582 | 0.583 | -4.983 |
| **52663-65-7** | 2,2',3,3',4,6,6'-Heptachlorobiphenyl | Training | 0.767 | 0.742 | -0.025 | -0.058 | 0.015 | 1 | 0 | 11.580 | 1.669 | 0.36624712 | 0.750 | -5.659 |
| **52663-66-8** | 2,2',3,3',4,5'-Hexachlorobiphenyl | Training | 0.431 | 0.651 | 0.220 | 0.507 | 0.012 | 1 | 0 | 11.956 | 1.612 | 0.37842764 | 0.694 | -10.120 |
| **52663-68-0** | 2,2',3,4',5,5',6-Heptachlorobiphenyl | Training | 0.756 | 0.799 | 0.043 | 0.099 | 0.016 | 1 | 0 | 11.731 | 1.707 | 0.34790638 | 0.778 | -5.960 |
| **52663-69-1** | 2,2',3,4,4',5',6-Heptachlorobiphenyl | Training | 0.790 | 0.768 | -0.022 | -0.052 | 0.016 | 1 | 0 | 11.856 | 1.669 | 0.34993075 | 0.778 | -6.091 |
| **52663-70-4** | 2,2',3,3',4,5',6'-Heptachlorobiphenyl | Training | 0.796 | 0.747 | -0.049 | -0.114 | 0.015 | 1 | 0 | 11.736 | 1.664 | 0.36743999 | 0.778 | -5.760 |
| **52663-71-5** | 2,2',3,3',4,4',6-Heptachlorobiphenyl | Training | 0.848 | 0.716 | -0.133 | -0.307 | 0.015 | 1 | 0 | 11.861 | 1.626 | 0.36946436 | 0.778 | -5.876 |

| | | | | | | | | | | | | | | |
|---|---|---|---|---|---|---|---|---|---|---|---|---|---|---|
| **52663-72-6** | 2,3',4,4',5,5'-Hexachlorobiphenyl | Prediction | 0.732 | 0.663 | -0.069 | -0.159 | 0.013 | 1 | 0 | 12.277 | 1.612 | 0.36709896 | 0.722 | -9.615 |
| **52663-74-8** | 2,2',3,3',4,5,5'-Heptachlorobiphenyl | Training | 0.854 | 0.732 | -0.122 | -0.283 | 0.015 | 1 | 0 | 11.865 | 1.664 | 0.35946864 | 0.750 | -5.806 |
| **52663-75-9** | 2,2',3,3',4,5,5',6'-Octachlorobiphenyl | Training | 0.820 | 0.916 | 0.096 | 0.223 | 0.020 | 1 | 0 | 11.688 | 1.716 | 0.33703975 | 0.889 | -11.709 |
| **52663-77-1** | 2,2',3,3',4,5,5',6,6'-Nonachlorobiphenyl | Training | 0.728 | 1.012 | 0.284 | 0.661 | 0.029 | 1 | 0 | 11.531 | 1.73 | 0.33143381 | 1.028 | -6.465 |
| **52663-78-2** | 2,2',3,3',4,4',5,6-Octachlorobiphenyl | Training | 0.641 | 0.804 | 0.163 | 0.377 | 0.020 | 1 | 0 | 11.799 | 1.635 | 0.36183404 | 0.889 | -4.784 |
| **52663-79-3** | 2,2',3,3',4,4',5,6,6'-Nonachlorobiphenyl | Prediction | 0.799 | 0.986 | 0.187 | 0.435 | 0.029 | 1 | 0 | 11.644 | 1.692 | 0.33003704 | 1.028 | -6.416 |
| **526-73-8** | 1,3,5-Trimethylbenzene | Training | -1.602 | -1.383 | 0.219 | 0.514 | 0.044 | 0 | 0 | 3.524 | 0.505 | 0.40875 | 0.000 | -3.688 |
| **52712-04-6** | 2,2',3,4,5,5'-Hexachlorobiphenyl | Training | 0.839 | 0.626 | -0.213 | -0.491 | 0.013 | 1 | 0 | 11.852 | 1.612 | 0.38472923 | 0.694 | -4.190 |
| **52712-05-7** | 2,2',3,4,5,5',6-Heptachlorobiphenyl | Prediction | 0.845 | 0.708 | -0.137 | -0.316 | 0.015 | 1 | 0 | 11.624 | 1.626 | 0.37912328 | 0.778 | -3.730 |
| **52744-13-5** | 2,2',3,3',5,6'-Hexachlorobiphenyl | Training | 0.724 | 0.716 | -0.008 | -0.019 | 0.013 | 1 | 0 | 11.642 | 1.655 | 0.37248067 | 0.722 | -8.942 |
| **52886-35-8** | 3-methyl-1-hexyl cyclohexane | Training | -1.181 | -0.740 | 0.440 | 1.031 | 0.039 | 0 | 0 | 1.000 | 0.199 | 0 | 0.146 | -2.835 |
| **53-19-0** | o,p'-DDD | Training | 0.380 | 0.712 | 0.331 | 0.770 | 0.025 | 1 | 0 | 6.040 | 1.431 | 0.40192854 | 0.472 | -5.118 |
| **5325-97-3** | 1,2,3,4,5,6,7,8- | Training | -0.893 | -1.045 | -0.152 | -0.357 | 0.039 | 0 | 0 | 3.470 | 0.781 | 0.33840278 | 0.125 | -3.984 |

| | | | | | | | | | | | | | | |
|---|---|---|---|---|---|---|---|---|---|---|---|---|---|---|
| | octahydrophenanthrene | | | | | | | | | | | | | |
| **53-70-3** | Dibenzo[a,h]anthracene | Training | -2.155 | -1.680 | 0.475 | 1.124 | 0.060 | 0 | 0 | 26.582 | 1.945 | 0.45 | 0.528 | -6.205 |
| **541-02-6** | Decamethylcyclopentasiloxane | Training | 0.532 | 0.847 | 0.316 | 0.858 | 0.287 | 0 | 0 | 1.000 | 0.452 | 0 | 1.944 | -74.624 |
| **544-76-3** | n-Hexadecane | Prediction | -0.314 | -0.641 | -0.327 | -0.765 | 0.038 | 0 | 0 | 1.000 | 0.127 | 0 | 0.056 | -55.784 |
| **55215-18-4** | 2,2',3,3',4,5-Hexachlorobiphenyl | Prediction | 0.839 | 0.582 | -0.257 | -0.593 | 0.013 | 1 | 0 | 11.857 | 1.569 | 0.39870988 | 0.694 | -4.081 |
| **556-67-2** | Octamethylcyclotetrasiloxane | Prediction | 0.602 | 0.105 | -0.497 | -1.181 | 0.068 | 0 | 0 | 1.000 | 0.417 | 0 | 0.944 | -56.561 |
| **55702-45-9** | 2,3,6-Trichlorobiphenyl | Training | 0.470 | 0.149 | -0.321 | -0.743 | 0.017 | 1 | 0 | 11.773 | 1.337 | 0.43449606 | 0.306 | -2.839 |
| **55712-37-3** | 2,3',4-Trichlorobiphenyl | Prediction | 0.556 | 0.254 | -0.303 | -0.700 | 0.014 | 1 | 0 | 12.329 | 1.38 | 0.42241875 | 0.444 | -4.609 |
| **5617-41-4** | n-heptyl cyclohexane | Training | -1.699 | -0.757 | 0.942 | 2.207 | 0.040 | 0 | 0 | 1.000 | 0.238 | 0 | 0.090 | -2.265 |
| **56-49-5** | 3-methylcholanthene | Training | -1.523 | -1.331 | 0.192 | 0.449 | 0.033 | 0 | 0 | 19.474 | 1.681 | 0.44094122 | 0.601 | -7.639 |
| **56-55-3** | Benzo[a]anthracene | Training | -2.301 | -1.815 | 0.487 | 1.144 | 0.048 | 0 | 0 | 23.672 | 1.603 | 0.45 | 0.347 | -5.702 |
| **56558-16-8** | 2,2',4,6,6'-Pentachlorobiphenyl | Training | 0.393 | 0.358 | -0.035 | -0.080 | 0.015 | 1 | 0 | 11.606 | 1.532 | 0.40106044 | 0.361 | -4.858 |
| **5707-44-8** | 4-ethyl-1,1'-biphenyl | Prediction | -1.745 | -1.489 | 0.256 | 0.594 | 0.024 | 0 | 0 | 12.296 | 0.995 | 0.44441358 | 0.313 | -3.010 |
| **57465-28-8** | 3,3',4,4',5-Pentachlorobiphenyl | Training | 0.581 | 0.441 | -0.140 | -0.323 | 0.012 | 1 | 0 | 12.527 | 1.517 | 0.40097756 | 0.583 | -5.242 |
| **581-40-8** | 2,3-Dimethylnaphthalene | Training | -1.921 | -1.397 | 0.523 | 1.220 | 0.030 | 0 | 0 | 10.705 | 0.871 | 0.43795352 | 0.167 | -43.252 |
| **60145-20-2** | 2,2',3,3',5-Pentachlorobiphenyl | Training | 0.761 | 0.505 | -0.256 | -0.592 | 0.011 | 1 | 0 | 11.799 | 1.56 | 0.40300193 | 0.583 | -4.843 |

| | | | | | | | | | | | | | | |
|---|---|---|---|---|---|---|---|---|---|---|---|---|---|---|
| **60145-23-5** | 2,2',3,4,4',5,6'-Heptachlorobiphenyl | Training | 0.836 | 0.810 | -0.025 | -0.059 | 0.020 | 1 | 0 | 11.858 | 1.631 | 0.35483556 | 0.889 | -5.555 |
| **60233-25-2** | 2,2',3,4',6'-Pentachlorobiphenyl | Training | 0.230 | 0.467 | 0.237 | 0.547 | 0.011 | 1 | 0 | 11.843 | 1.527 | 0.40229145 | 0.556 | -5.057 |
| **608-93-5** | Pentachlorobenzene | Training | -0.387 | 0.473 | 0.860 | 2.019 | 0.043 | 1 | 0 | 3.548 | 0.918 | 0.3436334 | 0.000 | -38.270 |
| **6117-97-1** | 4-methyl dodecane | Training | -1.114 | -0.824 | 0.290 | 0.680 | 0.042 | 0 | 0 | 1.000 | 0.088 | 0 | 0.111 | -3.143 |
| **62338-09-4** | 2,2,3-Trimethyl decane | Prediction | -1.181 | -0.799 | 0.382 | 0.896 | 0.046 | 0 | 0 | 1.000 | 0.009 | 0 | 0.222 | -2.035 |
| **629-50-5** | n-Tridecane | Training | -0.569 | -0.678 | -0.110 | -0.256 | 0.035 | 0 | 0 | 1.000 | 0.127 | 0 | 0.056 | -45.803 |
| **629-59-4** | n-Tetradecane | Training | 0.029 | -0.661 | -0.690 | -1.614 | 0.036 | 0 | 0 | 1.000 | 0.127 | 0 | 0.056 | -50.461 |
| **629-73-2** | 1-Hexadecene | Training | 0.183 | -1.133 | -1.316 | -3.108 | 0.055 | 0 | 0 | 1.083 | 0.167 | 0.36641337 | 0.056 | -55.784 |
| **66246-88-6** | 1H-1,2,4-Triazole, 1-[2-(2,4-Dichlorophenyl)pentyl]- | Training | -2.000 | -1.541 | 0.459 | 1.105 | 0.092 | 1 | 5 | 4.617 | 1.307 | 0.67916108 | 0.569 | -16.746 |
| **67129-08-2** | Metazachlor | Training | -4.494 | -3.203 | 1.291 | 3.119 | 0.098 | 0 | 7 | 4.692 | 2 | 0.60035435 | 0.715 | -4.971 |
| **68194-04-7** | 2,2',4,6'-Tetrachlorobiphenyl | Training | -0.854 | 0.296 | 1.150 | 2.661 | 0.015 | 1 | 0 | 11.814 | 1.475 | 0.40666638 | 0.333 | -7.174 |
| **68194-05-8** | 2,2',3,4',6-Pentachlorobiphenyl | Training | 0.303 | 0.476 | 0.173 | 0.399 | 0.012 | 1 | 0 | 11.801 | 1.565 | 0.38762451 | 0.500 | -5.282 |
| **68194-14-9** | 2,2',3,4,5',6-Hexachlorobiphenyl | Training | 0.799 | 0.620 | -0.180 | -0.415 | 0.013 | 1 | 0 | 11.739 | 1.617 | 0.38341533 | 0.667 | -4.254 |
| **68194-15-0** | 2,2',3,4,5,6'-Hexachlorobiphenyl | Training | -0.523 | 0.652 | 1.175 | 2.717 | 0.015 | 1 | 0 | 11.659 | 1.574 | 0.39808228 | 0.778 | -3.961 |
| **68194-16-1** | 2,2',3,3',4,5,6-Heptachlorobiphenyl | Prediction | 0.009 | 0.667 | 0.659 | 1.524 | 0.015 | 1 | 0 | 11.629 | 1.583 | 0.39107956 | 0.778 | -3.653 |
| **6975-98-0** | 2-methyl decane | Training | -1.444 | -0.824 | 0.620 | 1.455 | 0.042 | 0 | 0 | 1.000 | 0.088 | 0 | 0.111 | -3.142 |

| | | | | | | | | | | | | | | |
|---|---|---|---|---|---|---|---|---|---|---|---|---|---|---|
| **7012-37-5** | 2,4,4'-Trichlorobiphenyl | Prediction | 0.290 | 0.220 | -0.071 | -0.163 | 0.015 | 1 | 0 | 12.452 | 1.38 | 0.41623819 | 0.389 | -4.923 |
| **702-79-4** | 1,3-Dimethyladamantane | Training | -1.174 | -0.599 | 0.575 | 1.344 | 0.036 | 0 | 0 | 1.000 | 0.341 | 0 | 0.000 | -44.862 |
| **70356-09-1** | 1-(4-tert-butylphenyl)-3-(4-methoxyphenyl)propane-1,3-Dione | Training | -0.914 | -1.736 | -0.821 | -1.970 | 0.084 | 0 | 4 | 4.621 | 1.979 | 0.56084205 | 1.056 | -5.420 |
| **70362-45-7** | 2,2',3,6-Tetrachlorobiphenyl | Training | 0.568 | 0.310 | -0.259 | -0.598 | 0.014 | 1 | 0 | 11.664 | 1.47 | 0.41814577 | 0.389 | -3.566 |
| **70362-46-8** | 2,2',3,5-Tetrachlorobiphenyl | Prediction | 0.829 | 0.389 | -0.440 | -1.017 | 0.012 | 1 | 0 | 11.804 | 1.47 | 0.42017014 | 0.528 | -3.482 |
| **70424-68-9** | 2,3,3',4',5-Pentachlorobiphenyl | Training | 1.033 | 0.478 | -0.555 | -1.282 | 0.011 | 1 | 0 | 12.140 | 1.56 | 0.39559585 | 0.556 | -5.400 |
| **70424-70-3** | 2,3',4',5,5'-Pentachlorobiphenyl | Prediction | 0.380 | 0.523 | 0.143 | 0.330 | 0.012 | 1 | 0 | 12.087 | 1.56 | 0.39033517 | 0.611 | -4.903 |
| **7045-71-8** | 2-methyl undecane | Training | -1.292 | -0.822 | 0.470 | 1.103 | 0.042 | 0 | 0 | 1.000 | 0.088 | 0 | 0.111 | -3.532 |
| **7116-96-3** | 4-pentyl-1,1'-Biphenyl | Training | -1.174 | -1.370 | -0.196 | -0.456 | 0.026 | 0 | 0 | 10.753 | 0.995 | 0.43913206 | 0.340 | -3.036 |
| **71608-00-9** | 1-octylpyrene | Training | -1.108 | -1.568 | -0.460 | -1.074 | 0.033 | 0 | 0 | 20.215 | 1.479 | 0.43283919 | 0.486 | -3.439 |
| **717-74-8** | Benzene, 1,3,5-Tris(1-methylethyl)- | Prediction | -0.398 | -0.433 | -0.035 | -0.085 | 0.103 | 0 | 0 | 3.407 | 0.388 | 0.30805556 | 1.042 | -53.426 |
| **71888-89-6** | Diisoheptyl phthalate | Training | -2.509 | -2.726 | -0.217 | -0.528 | 0.107 | 0 | 6 | 3.096 | 1.313 | 0.56966446 | 0.694 | -94.461 |
| **72-43-5** | Methoxy chlor | Training | -0.969 | -1.961 | -0.992 | -2.332 | 0.047 | 0 | 4 | 5.764 | 1.504 | 0.47595165 | 0.653 | -73.385 |
| **72-54-8** | p,p'-DDD | Training | 0.602 | 0.956 | 0.354 | 0.829 | 0.042 | 1 | 0 | 6.173 | 1.431 | 0.37520186 | 0.472 | -62.499 |

| | | | | | | | | | | | | | | |
|---|---|---|---|---|---|---|---|---|---|---|---|---|---|---|
| **73575-52-7** | 2,3',4,5'-Tetrachlorobiphenyl | Prediction | 0.218 | 0.460 | 0.242 | 0.559 | 0.012 | 1 | 0 | 12.296 | 1.475 | 0.40040295 | 0.611 | -7.708 |
| **74472-33-6** | 2,3,3',6-Tetrachlorobiphenyl | Training | 0.720 | 0.364 | -0.356 | -0.822 | 0.012 | 1 | 0 | 11.817 | 1.47 | 0.41196521 | 0.472 | -3.798 |
| **74472-34-7** | 2,3,4',5-Tetrachlorobiphenyl | Training | 0.857 | 0.368 | -0.490 | -1.131 | 0.012 | 1 | 0 | 12.189 | 1.47 | 0.40658351 | 0.500 | -3.861 |
| **74472-36-9** | 2,3,3',5,6-Pentachlorobiphenyl | Training | 0.477 | 0.444 | -0.034 | -0.077 | 0.012 | 1 | 0 | 11.685 | 1.522 | 0.40635927 | 0.528 | -3.299 |
| **74472-37-0** | 2,3,4,4',5-Pentachlorobiphenyl | Training | 0.829 | 0.450 | -0.379 | -0.876 | 0.012 | 1 | 0 | 12.215 | 1.479 | 0.40097756 | 0.611 | -3.463 |
| **74472-38-1** | 2,3,4,4',6-Pentachlorobiphenyl | Prediction | 0.857 | 0.416 | -0.441 | -1.019 | 0.012 | 1 | 0 | 12.010 | 1.484 | 0.39762022 | 0.528 | -3.498 |
| **74472-42-7** | 2,3,3',4,4',6-Hexachlorobiphenyl | Training | 0.826 | 0.592 | -0.234 | -0.542 | 0.013 | 1 | 0 | 12.047 | 1.574 | 0.38663257 | 0.694 | -4.509 |
| **74472-44-9** | 2,3,3',4',5,6-Hexachlorobiphenyl | Training | 0.929 | 0.590 | -0.340 | -0.785 | 0.012 | 1 | 0 | 11.875 | 1.612 | 0.3846082 | 0.639 | -4.415 |
| **74472-48-3** | 2,2',3,4,4',6,6'-Heptachlorobiphenyl | Training | 0.928 | 0.701 | -0.227 | -0.525 | 0.014 | 1 | 0 | 11.669 | 1.636 | 0.35281119 | 0.694 | -5.714 |
| **74472-53-0** | 2,3,3',4,4',5,5',6-Octachlorobiphenyl | Training | 0.806 | 0.795 | -0.011 | -0.026 | 0.019 | 1 | 0 | 11.962 | 1.635 | 0.34848099 | 0.861 | -5.041 |
| **74487-85-7** | 2,2',3,4',5,6,6'-Heptachlorobiphenyl | Prediction | 0.884 | 0.799 | -0.085 | -0.196 | 0.017 | 1 | 0 | 11.621 | 1.674 | 0.3514973 | 0.806 | -5.618 |
| **779-02-2** | 9-Methylanthracene | Prediction | -2.398 | -1.759 | 0.639 | 1.489 | 0.030 | 0 | 0 | 18.248 | 1.237 | 0.44305556 | 0.236 | -4.039 |
| **781-17-9** | 4,5,9,10-Tetrahydropyrene | Training | -1.553 | -1.039 | 0.514 | 1.210 | 0.050 | 0 | 0 | 9.124 | 1.223 | 0.39875 | 0.111 | -56.126 |

| | | | | | | | | | | | | | | |
|---|---|---|---|---|---|---|---|---|---|---|---|---|---|---|
| **789-02-6** | 1-Chloro-2-(2,2,2-Trichloro-1-(4-chlorophenyl) ethyl)benzene (o,p'-DDT) | Training | 0.839 | 0.805 | -0.033 | -0.078 | 0.025 | 1 | 0 | 5.819 | 1.448 | 0.39632259 | 0.569 | -4.992 |
| **81-15-2** | Musk-xylene | Prediction | -0.210 | -0.799 | -0.589 | -1.525 | 0.213 | 0 | 3 | 4.638 | 0.882 | 0 | 1.618 | -6.160 |
| **81777-89-1** | Clomazone | Prediction | -3.699 | -0.893 | 2.806 | 6.662 | 0.065 | 1 | 4 | 3.099 | 1.238 | 0.45661226 | 0.476 | -3.035 |
| **821-95-4** | 1-undecene | Training | -1.409 | -1.201 | 0.208 | 0.489 | 0.049 | 0 | 0 | 1.152 | 0.167 | 0.37299195 | 0.056 | -41.173 |
| **83164-33-4** | Diflufenican | Training | -2.991 | -3.083 | -0.092 | -0.226 | 0.126 | 0 | 7 | 6.296 | 2.563 | 0.81116504 | 0.972 | -12.309 |
| **832-69-9** | 1-Methylphenanthrene | Training | -1.721 | -1.711 | 0.011 | 0.025 | 0.029 | 0 | 0 | 18.216 | 1.237 | 0.44609375 | 0.299 | -6.643 |
| **84-15-1** | o-Terphenyl | Prediction | -0.499 | -1.346 | -0.847 | -1.990 | 0.046 | 0 | 0 | 18.180 | 1.461 | 0.45 | 0.333 | -65.396 |
| **84632-59-7** | 3,6-Bis-(4-tert-butyl-phenyl)-2,5-dihydro-pyrrolo[3,4-c]pyrrole-1,4-dione | Training | -1.413 | -1.530 | -0.116 | -0.291 | 0.161 | 0 | 6 | 7.163 | 2.449 | 0.47522555 | 1.654 | -107.949 |
| **85-01-8** | Phenanthrene | Training | -1.122 | -1.847 | -0.725 | -1.693 | 0.035 | 0 | 0 | 18.554 | 1.261 | 0.45 | 0.125 | -3.984 |
| **872-05-9** | 1-decene | Training | -0.991 | -1.230 | -0.239 | -0.561 | 0.047 | 0 | 0 | 1.164 | 0.167 | 0.37505807 | 0.056 | -34.385 |
| **88671-89-0** | Myclobutanil | Training | -2.097 | -1.561 | 0.536 | 1.282 | 0.079 | 1 | 6 | 4.618 | 1.975 | 0.70261728 | 0.684 | -7.169 |
| **91-17-8** | t-decalin | Training | -0.068 | -0.633 | -0.566 | -1.322 | 0.035 | 0 | 0 | 1.000 | 0.349 | 0 | 0.000 | -34.486 |
| **91-20-3** | Naphthalene | Prediction | -2.301 | -1.530 | 0.771 | 1.800 | 0.034 | 0 | 0 | 10.721 | 0.919 | 0.45 | 0.000 | -34.486 |
| **91-57-6** | 2-Methylnaphthalene | Training | -2.000 | -1.600 | 0.400 | 0.932 | 0.028 | 0 | 0 | 10.744 | 0.895 | 0.44489796 | 0.083 | -3.391 |
| **92-06-8** | m-terphenyl | Training | -1.347 | -1.306 | 0.040 | 0.095 | 0.047 | 0 | 0 | 19.290 | 1.461 | 0.45 | 0.500 | -64.879 |
| **92-51-3** | Bicyclohexyl | Training | -0.770 | -0.503 | 0.266 | 0.620 | 0.030 | 0 | 0 | 1.000 | 0.349 | 0 | 0.139 | -44.916 |
| **933-12-0** | 3,5,5 Trimethyl cyclohexene | Training | -1.137 | -1.205 | -0.068 | -0.159 | 0.031 | 0 | 0 | 1.416 | 0.259 | 0.26944444 | 0.000 | -2.054 |

| | | | | | | | | | | | | | | |
|---|---|---|---|---|---|---|---|---|---|---|---|---|---|---|
| **93-46-9** | N,N′-Di-2-naphthyl-p-phenyleneDiamine | Training | -1.096 | -1.787 | -0.692 | -1.835 | 0.252 | 0 | 4 | 11.419 | 3.309 | 0.49340278 | 0.361 | 0.000 |
| **94361-06-5** | Cyproconazole | Training | -1.959 | -1.686 | 0.273 | 0.660 | 0.102 | 1 | 6 | 4.021 | 1.566 | 0.69527817 | 0.771 | -7.107 |
| **98-51-1** | 1-tert butyl-4-methyl benzene | Training | -1.018 | -1.133 | -0.115 | -0.270 | 0.040 | 0 | 0 | 3.323 | 0.45 | 0.37659722 | 0.354 | -1.549 |

**Table S4** – List of the excluded molecules.

| CAS | Chemical Name | SMILES | Log_BMF$_L$ Low Quality | Outliers info |
|---|---|---|---|---|
| **60207-90-1** | Propiconazole | CCCC1COC(O1)(Cn1cncn1)c1ccc(cc1Cl)Cl | -2.222 | out AD of Equation 1 |
| **70362-49-1** | 3,3',4,5-Tetrachlorobiphenyl | Clc1cccc(c1)c1cc(Cl)c(c(c1)Cl)Cl | -2.523 | out AD of Equation 1 |
| **10605-21-7** | Carbendazim | COC(=O)Nc1nc2c([nH]1)cccc2 | -3.745 | out AD of Equation 2 |
| **120068-36-2** | Fipronil sulfone | N#Cc1nn(c(c1S(=O)(=O)C(F)(F)F)N)c1c(Cl)cc(cc1Cl)C(F)(F)F | 0.777 | out AD of Equation 2 |
| **131860-33-8** | Azoxystrobin | COC=C(c1ccccc1Oc1ncnc(c1)Oc1ccccc1C#N)C(=O)OC | -3.347 | out AD of Equation 2 |
| **208465-21-8** | Mesosulfuron-methyl | COC(=O)c1ccc(cc1S(=O)(=O)NC(=O)Nc1nc(OC)cc(n1)OC)CNS(=O)(=O)C | -5.699 | out AD of Equation 2 |
| **2385-85-5** | Mirex | ClC12C(Cl)(Cl)C3(C4(C1(Cl)C1(C2(Cl)C3(C4(C1(Cl)Cl)Cl)Cl)Cl)Cl)Cl | 0.371 | out AD of Equation 2 |
| **2471-09-2** | OctacosamethylTridecasiloxane | C[Si](O[Si](O[Si](O[Si](O[Si](O[Si](O[Si](C)(C)C)(C)C)(C)C)(C)C)(C)C)(C)C)(O[Si](O[Si](O[Si](O[Si](O[Si](O[Si](C)(C)C)(C)C)(C)C)(C)C)(C)C)(C)C)C | -1.699 | out AD of Equation 2 |
| **2471-10-5** | Triacontamethyltetradecasiloxane | C[Si](O[Si](O[Si](O[Si](O[Si](O[Si](O[Si](C)(C)C)(C)C)(C)C)(C)C)(C)C)(C)C)(O[Si](O[Si](O[Si](O[Si](O[Si](O[Si](O[Si](C)(C)C)(C)C)(C)C)(C)C)(C)C)(C)C)(C)C)C | -1.699 | out AD of Equation 2 |
| **2652-13-3** | Eicosamethylnonasiloxane | C[Si](O[Si](O[Si](O[Si](O[Si](C)(C)C)(C)C)(C)C)(C)C)(O[Si](O[Si](O[Si](O[Si](C)(C)C)(C)C)(C)C)(C)C)C | -1.097 | out AD of Equation 2 |
| **34123-59-6** | Isoproturon | O=C(N(C)C)Nc1ccc(cc1)C(C)C | -3.211 | out AD of Equation 2 |
| **36734-19-7** | Iprodione | CC(NC(=O)N1CC(=O)N(C1=O)c1cc(Cl)cc(c1)Cl)C | -2.673 | out AD of Equation 2 |
| **50563-36-5** | Dimethachlor | COCCN(c1c(C)cccc1C)C(=O)CCl | -4.462 | out AD of Equation 2 |
| **541-01-5** | Hexadecamethylheptasiloxane | C[Si](O[Si](O[Si](O[Si](C)(C)C)(C)C)(C)C)(O[Si](O[Si](O[Si](C)(C)C)(C)C)(C)C)C | -1.398 | out AD of Equation 2 |
| **556-69-4** | Octadecamethyloctasiloxane | C[Si](O[Si](O[Si](O[Si](C)(C)C)(C)C)(C)C)(O[Si](O[Si](O[Si](O[Si](C)(C)C)(C)C)(C)C)(C)C)C | -0.796 | out AD of Equation 2 |
| **556-70-7** | Docosamethyldecasiloxane | C[Si](O[Si](O[Si](O[Si](O[Si](C)(C)C)(C)C)(C)C)(C)C)(O[Si](O[Si](O[Si](O[Si](O[Si](C)(C)C)(C)C)(C)C)(C)C)(C)C)C | -1.222 | out AD of Equation 2 |
| **79277-27-3** | Thifensulfuron-methyl | COc1nc(nc(n1)C)NC(=O)NS(=O)(=O)c1ccsc1C(=O)OC | -4.297 | out AD of Equation 2 |

**Table S5 –** List of the molecules outside the Applicability Domain (AD) of equations 1 and 3. CAS numbers, SMILES, Structures, chemicals common names, and information on the AD.

| CAS | SMILES | Structure | Common Name | out AD Eq.1 | out AD Eq.3 |
|---|---|---|---|---|---|
| **81-15-2** | O=N(=O)c1c(C)c(N(=O)=O)c(c(c1C(C)(C)C)N(=O)=O)C | 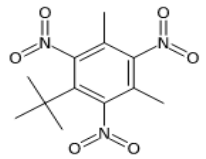 | Musk-xylene | **x** | **x** |
| **118-82-1** | CC(c1cc(Cc2cc(c(c(c2)C(C)(C)C)O)C(C)(C)C)cc(c1O)C(C)(C)C)(C)C | 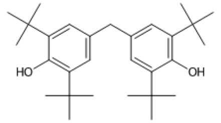 | Binox M | **x** | **x** |
| **120068-37-3** | N#Cc1nn(c(c1S(=O)C(F)(F)F)N)c1c(Cl)cc(cc1Cl)C(F)(F)F | 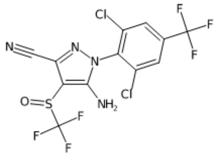 | Fipronil | **x** | **x** |
| **541-02-6** | C[Si]1(C)O[Si](C)(C)O[Si](C)(C)O[Si](O[Si](O1)(C)C)(C)C | 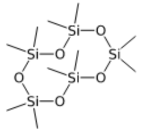 | Decamethylcyclopentasiloxane | **x** | **x** |
| **118-74-1** | Clc1c(Cl)c(Cl)c(c(c1Cl)Cl)Cl | 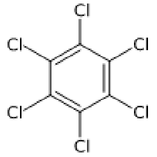 | Hexachlorobenzene | **x** | |

| | | | | | |
|---|---|---|---|---|---|
| **123-48-8** | CC(=CC(C)(C)C)CC(C)(C)C | 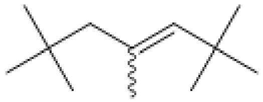 | 2,2,4,4,6,6 pentamethyl-3-heptene | **x** | |
| **1836-75-5** | Clc1ccc(c(c1)Cl)Oc1ccc(cc1)N(=O)=O | 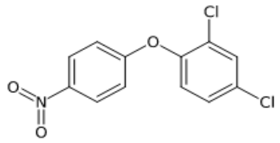  | 2,4-Dichloro-1-(4-nitrophenoxy) benzene | **x** | |
| **4390-04-9** | CC(CC(CC(C)(C)C)(C)C)CC(C)(C)C | 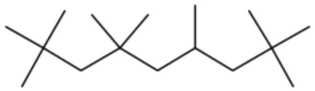 | 2,2,4,4,6,8,8-heptamethyl nonane | **x** | |
| **84632-59-7** | CC(c1ccc(cc1)c1[nH]c(=O)c2c1c(=O)[nH]c2c1ccc(cc1)C(C)(C)C)(C)C | 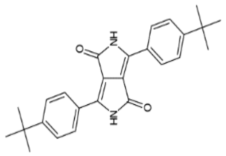 | 3,6-Bis-(4-tert-butyl-phenyl)-2,5-dihydro-pyrrolo[3,4-c]pyrrole-1,4-dione | | **x** |

| | | | | | |
|---|---|---|---|---|---|
| **112281-77-3** | Clc1ccc(c(c1)Cl)C(Cn1cncn1)C )F)(F)F | 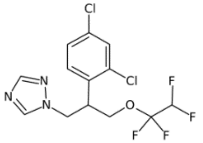 | Tetraconazole | | **x** |
| **43121-43-3** | O=C(C(C)(C)C)C(n1ncnc1)Oc 1ccc(cc1)Cl | 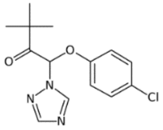 | Triadimefon | | **x** |
| **93-46-9** | c1ccc2c(c1)cc(cc2)Nc1ccc(cc1 )Nc1ccc2c(c1)cccc2 | 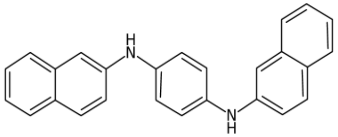 | N,N′-Di-2-naphthyl-p-phenyleneDiamine | | **x** |
| **83164-33-4** | Fc1ccc(c(c1)F)NC(=O)c1cccn c1Oc1cccc(c1)C(F)(F)F | 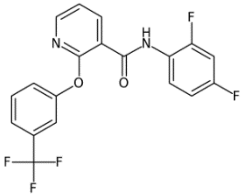 | Diflufenican | | **x** |
| **16958-92-2** | CCCCCCCCCCCCCOC(=O) CCCCC(=O)OCCCCCCCCC CCCC | 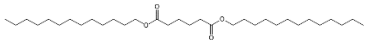 | Diisotridecyl adipate | | **x** |
